# Definition of the Spatial Propagator and Implications for Magnetic Field Properties

**DOI:** 10.1007/s11207-019-1452-4

**Published:** 2019-06-12

**Authors:** Justin K. Edmondson, Pascal Démoulin

**Affiliations:** 1 Department of Climate and Space Science and Engineering, University of Michigan, 2455 Hayward St., Ann Arbor, MI 48109 USA; 2 LESIA, Observatoire de Paris, Université PSL, CNRS, Sorbonne Université, Univ. Paris Diderot, Sorbonne Paris Cité, 5 place Jules Janssen, 92195 Meudon, France

**Keywords:** Magnetic fields, corona, Corona, structures

## Abstract

We present a theoretical framework to analyze the 3D coronal vector magnetic-field structure. We assume that the vector magnetic field exists and is *a priori* smooth. We introduce a generalized connectivity phase space associated with the vector magnetic field in which the basic elements are the *field line* and its linearized variation: the *Spatial Propagator*. We provide a direct formulation of these elements in terms of the vector magnetic field and its spatial derivatives, constructed with respect to general curvilinear coordinates and the equivalence class of general affine parameterizations. The Spatial Propagator describes the geometric organization of the local bundle of field lines, equivalent to the kinematic deformation of a propagated volume tied to the bundle. The Spatial Propagator’s geometric properties are characterized by dilation, anisotropic stretch, and rotation. Extreme singular values of the Spatial Propagator describe quasi-separatrix layers (QSLs), while true separatrix surfaces and separator lines are identified by the vanishing of one and two singular values, respectively. Finally, we show that, among other possible applications, the squashing factor [$Q$] is easily constructed from an analysis of particular sub-matrices of the Spatial Propagator.

## Introduction

Geometry describes the measurable lengths and angles associated with a system’s configuration, whereas topology is concerned with those properties preserved under continuous deformation. Over the past several decades the importance of the geometric and topological features to fluid and plasma dynamics has become increasingly clear (see, *e.g.*, Moffatt *et al.*, 1991; Arnold and Khesin, 1998; Ricca, 2001). Algebraic and geometric analyses of magnetic and hydrodynamic structures have provided significant development in the context of general plasma equilibria (*e.g.* Moffatt, 1985, 1986), stability (*e.g.* Bulanov and Sasorov, 1978; Syrovatskii, 1981; Bulanov *et al.*, 1999), reconnection (Hesse and Schindler, 1988; Schindler, Hesse, and Birn, 1988; Ruzmaikin and Akhmetiev, 1994; Bulanov *et al.*, 2002; Pontin *et al.*, 2005), heating, and wave generation (Ruzmaikin and Berger, 1998). In particular, geometric and topological analyses of the solar coronal magnetic field have focused on understanding heating and dynamics (Antiochos, 1987; Berger, 1994; Priest, Longcope, and Heyvaerts, 2005), as well as investigations into the eruptive phenomenology of flares (Mandrini *et al.*, 1995; Démoulin *et al.*, 1997; Titov and Démoulin, 1999; Aulanier *et al.*, 2000) and coronal mass ejection (CME) initiation (Antiochos, DeVore, and Klimchuck, 1999; Lynch *et al.*, 2008; Lynch and Edmondson, 2013).

The build-up, storage, transport, and subsequent release of magnetic energy in the low-$\beta $ solar corona is widely accepted as the basic requirement for solar coronal heating, the origin and generation of the solar wind, as well as eruptive phenomenology and space-weather prediction (see, *e.g.*, Klimchuk, 2006, for a review). It is the geometric and topological features of the coronal magnetic field that govern these dynamics in the low-$\beta $ coronal plasma environment (see Longcope, 2005, for a review). In general, non-trivial geometric and topological features such as null-point structure (Lau and Finn, 1990; Parnell *et al.*, 1996), separator lines (Longcope and Cowley, 1996), bald patches (Seehafer, 1986; Wolfson, 1989; Low, 1992; Titov, Priest, and Démoulin, 1993), separatrix surfaces (Low, 1987; Somov, 1992; Priest, Heyvaerts, and Title, 2002), and quasi-separatrix layers (QSLs: Priest and Démoulin, 1995; Démoulin *et al.*, 1996, 1997; Titov, 1999; Milano *et al.*, 1999) appear ubiquitously in the coronal magnetic field. In fact, the current state of global solar-wind generation models (see, *e.g.*, Abbo *et al.*, 2016, for a recent review), differ in the extent and complexity of these geometric and topological structures within the coronal magnetic field (*e.g.* Wang and Sheeley, 1990; Fisk, Schwadron, and Zurbuchen, 1998; Arge and Pizzo, 2000; Fisk, 2003; Cranmer, van Ballegooijen, and Edgar, 2007; Antiochos *et al.*, 2011; Antiochos, 2013).

Since the vector magnetic field is reference-frame dependent, so too is the magnetic-connectivity description (Hornig and Schindler, 1996); in fact, the entire concept of a magnetic-field line is not Lorentz invariant (Hornig, 1997). However, in a fixed reference frame, the geometric and topological features of the magnetic field constrain the dynamics. In general, the geometric features and topological constraints of the magnetic field are primarily important to understand where and how energy is stored and released in low-$\beta $ plasma environments such as the solar corona. In the presence of resistive (non-ideal) physics, field-line connectivity topology is no longer preserved, although separatrix structures remain. Separatrix surfaces and QSLs are associated with electric-current-sheet formation and reconnection, and therefore it is the geometry of these structures that constrain the storage of magnetic energy throughout the coronal volume, and determine both the location and the ability to release free energy (see the references above). In particular, QSL locations are related to observations of sudden flare brightening in H$\alpha $ for a wide variety of solar-flare phenomenology such as circular ribbon flares, two ribbon flares, and twisted flux rope (sigmoidal active region) morphologies (see, *e.g.*, Janvier, 2017, and the references therein). Furthermore, separatrix surfaces and QSLs are the boundaries dividing regions of disparate connectivity in complex, multi-polar coronal structures. Hence, QSLs feature prominently in the rapid reorganization of the vector magnetic field and subsequent energy release in CME initiation mechanisms (see, *e.g.*, Aulanier, Démoulin, and Grappin, 2005; Effenberger *et al.*, 2011; Janvier *et al.*, 2014; Schmieder, Aulanier, and Vršnak, 2015; Lynch *et al.*, 2016, and the references therein).

The features of the magnetic connectivity map such as helicity, separatrix and QSL structures, and their effect on the system dynamics find their most explicit representations in terms of the algebraic and geometric descriptions of the field line and the field-line bundle. In other words, the constraints on the system dynamics become transparent when cast in terms of the geometric structure and topological invariants of the field-line bundle and its behavior. Numerical magnetohydrodynamic (MHD) methodologies allow one to explore the locus of system dynamics for various magnetic-field models, thermodynamic heating models, and boundary conditions. While the MHD method (where applicable) is correct, the MHD equations and numerical solutions are often opaque to these explicit geometric structures (*e.g.* QSLs, separatrices, and separators) and their behavior. Moreover, many numerical difference schemes employ linear interpolation, which has the potential to limit these codes as an accurate representation of the system, especially in the vicinity of sharp gradients and other small-scale structures. Effectively, linear interpolation shifts the problem of the small-scale dynamics from the physical quantities on to finer and more complex numerical-grid resolutions. This is an extremely popular approach to numerical/computational solar- and space-plasma physics, but arguably leads to spurious effects (see, *e.g.*, Edmondson, 2012, for a discussion); we offer no judgment regarding the veracity of these approaches. This perspective, however, requires the development of new mathematical tools/description/framework to analyze the geometric organization of the magnetic-field connectivity map.

The purpose of this article is the introduction, development, and presentation of a generalized connectivity phase space of field-line geometry associated to the vector magnetic field, in which the geometric structure and topological constraints are made explicit. This formalism does not alter the physics of Maxwell’s equations, or MHD, but it is a fundamentally different framework that describes and analyzes vector magnetic fields. The basic assumptions of this framework are:

i) the vector magnetic field is the *primary (observable)* quantity that satisfies some standard physical evolutions (*e.g.* Maxwell’s equations, MHD induction, *etc.*), and the connectivity map is the *secondary (derived)* quantity; and
ii) while large gradients may exist, the vector magnetic field is *a priori* smooth everywhere.

Under these assumptions, we derive a generally covariant measure of the local field geometry, called the *Spatial Propagator*, from the linearized variation of a field line. We demonstrate that the Spatial Propagator characterizes the geometric organization of a local bundle of field lines. Moreover, we identify topological invariants derived from the Spatial Propagator, as well as demonstrate the proper limiting connection to QSLs, separatrix surfaces, and separator lines. Beyond the limiting cases, the inclusion and analysis of existing and/or generated singular structures within the vector field are outside the scope of the present work.

The roadmap for this article is as follows: In Section 2 we lay out a precise mathematical definition for the field lines of a vector field (Section 2.1). We introduce the Spatial Propagator (Section 2.2) as a generalized spatial variation of an entire field-line solution, which describes the local bundle of field lines. Furthermore, we derive a direct relation between the Spatial Propagator and the local gradient of the vector field (Section 2.3), and hence the precise mathematical formulation of the geometric phase space, consisting of the integral curve solutions and their associated Spatial Propagators. Finally, we discuss the various representations of the Spatial Propagator (Section 2.4): covariance with respect to local curvilinear coordinates, and equivalence with respect to affine transformations of the coordinate defined along the field line.

In Section 3 we characterize the geometric organization (dilation, rotation, anisotropic stretch, and connectivity gradient) of a local bundle of field lines using a mathematically equivalent kinematic analysis of a volume propagated along and deformed by the bundle. We explore the vector-field geometry using the Spatial Propagator in terms of volumetric dilation (Section 3.1), from which we identify a topological invariant measure that reflects the divergence-free condition of physical magnetic fields. We demonstrate that the singular values and singular vectors of the Spatial Propagator characterize the anisotropic stretch and rigid-body rotation deformations of the geometry (Section 3.2). Lastly (Section 3.3), we identify quasi-separatrix structures directly from the Spatial Propagator as extreme kinematic deformations, and we demonstrate the construction of the $Q$-factor (see, *e.g.*, Titov, Hornig, and Démoulin, 2002; Titov, 2007) by a simple example.

We close with a description of other potential applications of the Spatial Propagator in Section 4. The major objects and main equations of this framework are summarized in Table 1.

**Table 1:** Summary of major objects and main equations of this framework.

Symbol	Quantity name	Equations	Section
***B***(***r***)	Vector field	2	2.1
*λ*	Connectivity parameter	2	2.1
$\boldsymbol{r}_{0}$	Reference point	3	2.1
$\boldsymbol{r} ( \lambda , \boldsymbol{r}_{0} )$	Field line (for single fixed $\boldsymbol{r}_{0}$)	2	2.1
$\boldsymbol{r} ( \lambda , \boldsymbol{r}_{0} )$	Congruence (for all $\boldsymbol{r}_{0} \in \Omega _{0} \subset \mathbb{R}^{3}$)	2	2.1
∇***B***(***r***)	Covariant differential vector field matrix	15, 21	2.2, 2.4
$\mathbf{F} ( \lambda , \boldsymbol{r}_{0} )$	Spatial Propagator	11, 16	2.2
$\boldsymbol{v} ( \lambda , \boldsymbol{r}_{0} )$	Propagated shift vector	12	2.2
$\boldsymbol{h} = \boldsymbol{v} ( 0 , \boldsymbol{r}_{0} )$	Reference shift vector	10, 12	2.2
${\mathrm{d}} \Omega ( \lambda , \boldsymbol{r}_{0} )$	Signed differential volume element	29	3.1
$\Omega ( \lambda , \boldsymbol{r}_{0} )$	Total propagated volume	31	3.1
$\sigma _{\alpha } ( \lambda , \boldsymbol{r}_{0} )$	Singular values of $\mathbf{F} ( \lambda , \boldsymbol{r}_{0} )$	37	3.2
${\hat{\boldsymbol{l}}}_{\alpha } ( \lambda , \boldsymbol{r}_{0} )$, ${\hat{\boldsymbol{r}}}_{\alpha } ( \lambda , \boldsymbol{r}_{0} )$	Left-, right-singular vectors of $\mathbf{F} ( \lambda , \boldsymbol{r}_{0} )$	42	3.2
$\mathbf{V} ( \lambda , \boldsymbol{r}_{0} )$, $\mathbf{U} ( \lambda , \boldsymbol{r}_{0} )$	Left-, right-stretch matrices	40, 46	3.2
$\mathbf{R} ( \lambda , \boldsymbol{r}_{0} )$	Rotation matrix	41, 46	3.2
$Q ( \lambda , \boldsymbol{r}_{0} )$	Squashing factor [*Q*-Value]	56, 60	3.3

## The Integral Curve Description of Vector Field Geometry

Let $M \subseteq \mathbb{R}^{3}$ be a subset of three-dimensional space, possibly with boundary $\partial M$. Let $\boldsymbol{B} \left ( \boldsymbol{r} \right )$ be a vector field for all positions $\boldsymbol{r} \in M$. This field satisfies some set of dynamic evolution equations for some known initial and boundary conditions. Written with respect to a Cartesian^1^ basis $\lbrace \hat{\boldsymbol{e}}_{x}, \hat{\boldsymbol{e}}_{y}, \hat{\boldsymbol{e}}_{z} \rbrace $, the vector field $\boldsymbol{B} \left ( \boldsymbol{r} \right )$ has component functions,

1 $$\begin{aligned} \begin{aligned} \boldsymbol{B} ( \boldsymbol{r} ) \cdot \hat{ \boldsymbol{e}} _{x} &= ( \boldsymbol{B} \vert _{\boldsymbol{r}} )^{x} = B^{x} ( x, y, z ), \\ \boldsymbol{B} ( \boldsymbol{r} ) \cdot \hat{\boldsymbol{e}} _{y} &= ( \boldsymbol{B} \vert _{\boldsymbol{r}} )^{y} = B^{y} ( x, y, z ), \\ \boldsymbol{B} ( \boldsymbol{r} ) \cdot \hat{\boldsymbol{e}} _{z} &= ( \boldsymbol{B} \vert _{\boldsymbol{r}} )^{z} = B^{z} ( x, y, z ), \end{aligned} \end{aligned}$$

where $\boldsymbol{r}$ is the spatial position with coordinates $\lbrace x, y, z \rbrace $.

In general, the vector field $\boldsymbol{B} \left ( \boldsymbol{r} \right )$ is time-dependent, and therefore, strictly speaking, so too are the integral curves (Section 2.1), and the Spatial Propagator (Section 2.2), as well as all other objects constructed therefrom. For ease of notation, throughout this work we suppress the functional time-dependence of all quantities; this may be interpreted as analyzing the system at a fixed time, or for very-low frequency dynamics, $f \ll c/L$ where $c$ is the characteristic speed of communication, and $L$ is a characteristic length scale. The dynamical description of the integral curves (Section 2.1), the Spatial Propagator (Section 2.2), *etc.* require a treatment of the full four-dimensional electromagnetic-field tensor (Jackson, 1999, Section 11.9), which is outside the scope of this work.

### The Integral Curves of a Vector Field

In differential equation theory, a general vector field $\boldsymbol{B} \left ( \boldsymbol{r} \right )$ is everywhere tangent to a set of integral curves $\boldsymbol{r} \left ( \lambda , \boldsymbol{r}_{0} \right )$ that satisfy the initial value problem

2 $$\begin{aligned} \frac{\partial \boldsymbol{r} ( \lambda , \boldsymbol{r}_{0} )}{ \partial \lambda } =& \boldsymbol{B} ( \boldsymbol{r} ), \end{aligned}$$

3 $$\begin{aligned} \boldsymbol{r} ( 0 , \boldsymbol{r}_{0} ) =& \boldsymbol{r} _{0}. \end{aligned}$$

The family of integral curve solutions $\boldsymbol{r} \left ( \lambda , \boldsymbol{r}_{0} \right )$ are parametrized by a real number $\lambda \in \mathbb{R}$, referred to as the *connectivity parameter*, and a three-component vector $\boldsymbol{r}_{0} \in M$, referred to as the *reference point*. A single integral curve, as a particular solution to Equations 2 and 3, is identified by a single fixed reference point $\boldsymbol{r}_{0}$. The connectivity parameter $\lambda $ denotes the *distance per unit field strength* along a particular solution curve issuing from a particular reference point. We note that the rate of change with respect to $\lambda $ is simply the directional derivative along the vector field, which may be written with respect to the coordinate representation

4 $$\begin{aligned} \frac{\partial }{\partial \lambda } \equiv \boldsymbol{B} ( \boldsymbol{r} ) \cdot \nabla . \end{aligned}$$

The integral curve solutions to Equations 2 and 3 represent position vectors $\boldsymbol{r} = \boldsymbol{r} ( \lambda , \boldsymbol{r}_{0} )$, the components of which are functions of $( \lambda , \boldsymbol{r} _{0} )$; written with respect to a Cartesian basis $\lbrace \hat{\boldsymbol{e}}_{x}, \hat{\boldsymbol{e}}_{y}, \hat{\boldsymbol{e}} _{z} \rbrace $

5 $$\begin{aligned} \begin{aligned} \boldsymbol{r} ( \lambda , \boldsymbol{r}_{0} ) \cdot \hat{\boldsymbol{e}}_{x} &= ( \boldsymbol{r} \vert _{\lambda , \boldsymbol{r}_{0}} )^{x} = x ( \lambda , x_{0} , y_{0} , z_{0} ), \\ \boldsymbol{r} ( \lambda , \boldsymbol{r}_{0} ) \cdot \hat{ \boldsymbol{e}}_{y} &= ( \boldsymbol{r} \vert _{\lambda , \boldsymbol{r}_{0}} )^{y} = y ( \lambda , x_{0} , y_{0} , z_{0} ), \\ \boldsymbol{r} ( \lambda , \boldsymbol{r}_{0} ) \cdot \hat{ \boldsymbol{e}}_{z} &= ( \boldsymbol{r} \vert _{\lambda , \boldsymbol{r}_{0}} )^{z} = z ( \lambda , x_{0} , y_{0} , z_{0} ). \end{aligned} \end{aligned}$$

The integral curve solutions $\boldsymbol{r} ( \lambda , \boldsymbol{r}_{0} )$ are known by various names depending on the nature and interpretation of the differential equations and vector field. In physics, integral curves for electric and magnetic fields are known as *field lines*, whereas the integral curves for a velocity field are called *streamlines*. In general dynamical systems theory, the integral curves of the governing differential equation system are referred to as *trajectories* or *orbits*.

**Figure 1:**
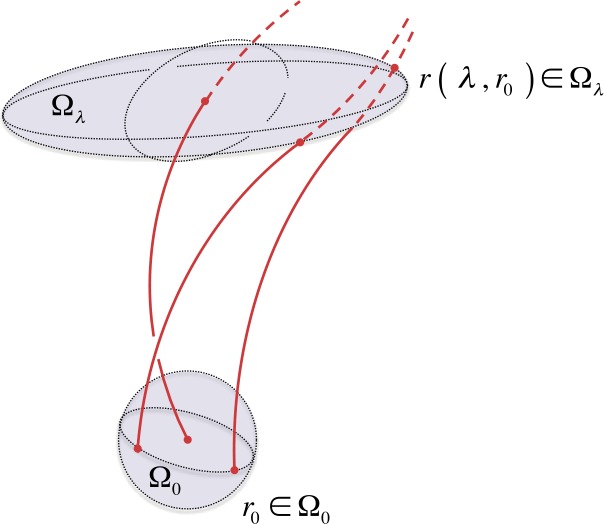
Illustration of a general congruence, *viz*. field lines and reference points. A particular field line $\boldsymbol{r} ( \lambda , \boldsymbol{r}_{0} )$ is associated with a single fixed reference point $\boldsymbol{r}_{0}$. A congruence is defined by a set of reference points in some neighborhood $\boldsymbol{r}_{0} \in \Omega _{0} \subseteq \mathbb{R}^{3}$.

Since we find application to solar magnetic fields, throughout this article we use *field line* to denote a particular solution to Equations 2 and 3 with fixed $\boldsymbol{r}_{0}$. Moreover, a *flux tube* is a *congruence of integral curves* (or simply a *congruence*); that is a local bundle of field lines defined by a particular set of reference points in some neighborhood $\boldsymbol{r}_{0} \in \Omega _{0} \subseteq M$ (see Figure 1).

We make a few observations and define some nomenclature regarding the field lines in physical systems of interest. The reference point $\boldsymbol{r}_{0}$ is a free parameter, typically chosen on the system boundary, or to coincide with some known initial state within the system interior; for example, in a magnetized plasma this choice typically coincides with a highly-conductive parcel of plasma material. The $\lambda = 0$ datum defined by the reference point $\boldsymbol{r}_{0} = \boldsymbol{r} ( 0, \boldsymbol{r}_{0} )$ is typically referred to as the *launch footpoint* in magnetic systems. The point $\boldsymbol{r} = \boldsymbol{r} ( L, \boldsymbol{r}_{0} )$ for a *finite* $\lambda = L$, corresponding to the final point of integration of Equations 2 and 3 is often referred to as the *target footpoint* in magnetic systems; typically, the target footpoint is where the integral curves crosses the system boundary, or encounters a singular structure in the vector field.

The congruence of integral curves of a smooth vector field represents an equivalence class under general affine transformations of the connectivity parameter of the form $\lambda \mapsto \ell ( \lambda ) = f ( \boldsymbol{r} ) \lambda + b$, where $f ( \boldsymbol{r} )$ is a smooth, positive definite, scalar-valued function,^2^ and $b \in \mathbb{R}$ an arbitrary constant.^3^ The re-parametrized flow $\boldsymbol{r} ( \ell , \boldsymbol{r}_{b} )$ satisfies Equations 2 and 3 for vector field $\boldsymbol{X} ( \boldsymbol{r} ) = \boldsymbol{B} ( \boldsymbol{r} ) / f ( \boldsymbol{r} )$ and reference condition $\boldsymbol{r}_{b}$; that is,

6 $$\begin{aligned} \frac{\partial \boldsymbol{r} ( \ell , \boldsymbol{r}_{0} )}{ \partial \ell } =& \boldsymbol{X} ( \boldsymbol{r} ), \end{aligned}$$

7 $$\begin{aligned} \boldsymbol{r} ( b , \boldsymbol{r}_{0} ) =& \boldsymbol{r} _{b}. \end{aligned}$$

A simple application of this $\lambda \mapsto \ell ( \lambda )$ re-parametrization is the analysis of the $\boldsymbol{B}$ field in a system with boundaries $\partial M$. One has the freedom to choose the function $f ( \boldsymbol{r} )$ (and constant $b = 0$) in order that the values $\ell = 0$ and $\ell = 1$ coincide with the initial and final points of the field lines taken at the boundary surfaces, $\boldsymbol{r}_{0} \in \partial M$ and $\boldsymbol{r} ( 1, \boldsymbol{r}_{0} ) \in \partial M$; typically at the photosphere for solar coronal applications. With $\ell = 0$ and $\ell = 1$ as boundary values, the connectivity parameter $\ell $ is not the physical (dimensional) arc length, but rather a normalized dimensionless arc-length parameter.

As a second application, consider $f ( \boldsymbol{r} ) = \vert \boldsymbol{B} ( \boldsymbol{r} ) \vert $; assuming $\vert \boldsymbol{B} ( \boldsymbol{r} ) \vert \ne 0$. The vector field $\boldsymbol{X} ( \boldsymbol{r} ) = \frac{\boldsymbol{B} ( \boldsymbol{r} )}{\vert \boldsymbol{B} ( \boldsymbol{r} ) \vert } = \boldsymbol{b} ( \boldsymbol{r} )$ is well defined and is identified with the unit magnetic-field direction. For simplicity and without loss of generality, we may set the constant $b = 0$. Under this transformation, the system of Equations 2 and 3 becomes

8 $$\begin{aligned} \frac{\partial \boldsymbol{r} ( \ell , \boldsymbol{r}_{0} )}{ \partial \ell } =& \boldsymbol{b} ( \boldsymbol{r} ), \end{aligned}$$

9 $$\begin{aligned} \boldsymbol{r} ( 0 , \boldsymbol{r}_{0} ) =& \boldsymbol{r} _{0} \end{aligned}$$

In this example, the connectivity parameter $\ell $ represents the physical arc length along the magnetic-field line. We refer to the representation constructed from the unit magnetic-field direction vector field and in which the connectivity parameter represents the physical arc length along the field line issuing from the reference point, as the *arc-length representation*.

### The Spatial Propagator: A Local Congruence of Integral Curves

The relative geometry of a local congruence (*i.e.* dilation, anisotropic stretch, and rotation of the bundle of curves) is completely described by examining a field line under a spatial variation of the reference point $\boldsymbol{r}_{0} \mapsto \boldsymbol{r}_{0} + \boldsymbol{h}$. We denote the spatial variation of the reference point by the *reference shift vector* $\boldsymbol{h}$.

Recall that a fixed reference point $\boldsymbol{r}_{0}$ is equivalent to a single field line $\boldsymbol{r} ( \lambda , \boldsymbol{r}_{0} )$; hence, when considering solutions $\boldsymbol{r} ( \lambda , \boldsymbol{r}_{0} )$ to Equations 2 and 3 for a set of $\boldsymbol{r}_{0} \in \Omega _{0}$, a variation in the reference point $\boldsymbol{r}_{0} \to \boldsymbol{r} _{0} + \boldsymbol{h}$ is equivalent to a variation of the *entire* field line. To see this, consider two spatially neighboring field-line solutions within the congruence, respectively: $\boldsymbol{r} ( \lambda , \boldsymbol{r}_{0} )$ and $\boldsymbol{r} ( \lambda , \boldsymbol{r}_{0} + \boldsymbol{h} )$. We may relate the component functions of the neighboring field lines by a Taylor expansion, such that for all $\lambda $ and $\vert \boldsymbol{h} \vert \ll h_{m}$,

10 $$ ( \boldsymbol{r} \vert _{\lambda , \boldsymbol{r}_{0} + \boldsymbol{h}} )^{i} - ( \boldsymbol{r} \vert _{\lambda , \boldsymbol{r}_{0}} )^{i} = \sum _{j} \frac{ \partial ( \boldsymbol{r} \vert _{\lambda , \boldsymbol{r}_{0}} ) ^{i}}{\partial \boldsymbol{r}_{0}{}^{j}} \boldsymbol{h}^{j} + O \bigl( \vert \boldsymbol{h} \vert ^{2} \bigr), $$

where $h_{m}$ is a local characteristic length scale defined by comparing the magnitude of the first-order with the higher-order variational terms (see Appendix A).

Equation 10 describes the local organization of all field lines with reference points within an initial volume $\Omega _{0}$ of characteristic size $\vert \Omega _{0} \vert ^{1/3} \ll h_{m}$; that is to say, the congruence is *local* with respect to reference points $\boldsymbol{r}_{0} + \boldsymbol{h} \in \Omega _{0}$ with $\vert \boldsymbol{h} \vert \ll h_{m}$.

We remark, there is an implicit assumption in Equation 10 that the magnetic vector field $\boldsymbol{B} ( \boldsymbol{r} )$ is described by smooth component functions everywhere within the domain. In future work, we will explore the consequences of relaxing this assumption.

We define this first-order variation of the field line $\boldsymbol{r} ( \lambda , \boldsymbol{r}_{0} )$ with respect to a spatial variation in the reference point $\boldsymbol{r}_{0}$ to be the *Spatial Propagator* $\mathbf{F} ( \lambda , \boldsymbol{r} _{0} )$. Then for all $\lambda $, the variational derivative may be represented as a $3 \times 3$ matrix whose component functions are simply the derivatives of Equation 5 with respect to the reference point components,

11 $$\begin{aligned} ( \mathbf{F} \vert _{\lambda , \boldsymbol{r}_{0}} ) ^{i}{}_{j} \equiv & \frac{\partial ( \boldsymbol{r} \vert _{\lambda , \boldsymbol{r}_{0}} ){}^{i}}{\partial \boldsymbol{r}_{0}{}^{j}} = \left ( \textstyle\begin{array} {c@{\quad }c@{\quad }c} \frac{\partial x}{\partial x_{0}} & \frac{\partial x}{\partial y_{0}} & \frac{\partial x}{\partial z_{0}} \\ \frac{\partial y}{\partial x_{0}} & \frac{\partial y}{\partial y_{0}} & \frac{\partial y}{\partial z_{0}} \\ \frac{\partial z}{\partial x_{0}} & \frac{\partial z}{\partial y_{0}} & \frac{\partial z}{\partial z_{0}} \end{array}\displaystyle \right ). \end{aligned}$$

Note that, at $\lambda = 0$, the field line reduces to the reference point $\boldsymbol{r} ( 0 , \boldsymbol{r}_{0} ) = \boldsymbol{r}_{0}$, and similarly $\boldsymbol{r} ( 0 , \boldsymbol{r}_{0} + \boldsymbol{h} ) = \boldsymbol{r}_{0} + \boldsymbol{h}$. Hence, Equation 10 reduces to the definition of the reference shift vector $\boldsymbol{r} ( 0, \boldsymbol{r}_{0} + \boldsymbol{h} ) - \boldsymbol{r} ( 0, \boldsymbol{r}_{0} ) = \boldsymbol{h}$, and Equation 11 at $\lambda = 0$ is simply the $3 \times 3$ identity matrix, $( \mathbf{F} \vert _{0 , \boldsymbol{r}_{0}} ){}^{i}{}_{j} = \delta ^{i}{}_{j}$; where $\delta ^{i}{}_{j}$ is the Kronecker delta.

**Figure 2:**
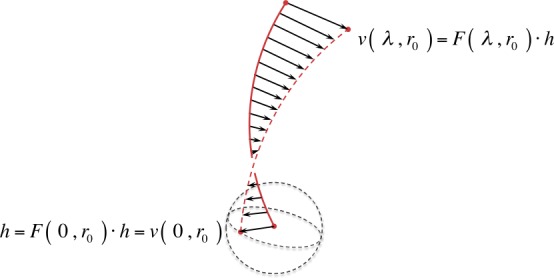
Illustration of the action of the Spatial Propagator. The spatial variation of a particular field line (*solid red*) in the direction $\boldsymbol{r}_{0} \rightarrow \boldsymbol{r}_{0} + \boldsymbol{h}$ is indicated by the *dashed red line*.

The Spatial Propagator $\mathbf{F} ( \lambda , \boldsymbol{r}_{0} )$ may be considered to be the generalized gradient of an entire field line $\boldsymbol{r} ( \lambda , \boldsymbol{r}_{0} )$ issuing from the reference point $\boldsymbol{r}_{0}$ for all $\lambda $. The difference vector $\boldsymbol{v} ( \lambda , \boldsymbol{r}_{0} )$ represents the spatial shift at each $\lambda $ along the particular field line $\boldsymbol{r} ( \lambda , \boldsymbol{r}_{0} )$ to the corresponding point at $\lambda $ along the neighboring field line $\boldsymbol{r} ( \lambda , \boldsymbol{r}_{0} + \boldsymbol{h} )$ (see Figure 2); the components of $\boldsymbol{v} ( \lambda , \boldsymbol{r}_{0} )$ are given by

12 $$ ( \boldsymbol{v} \vert _{\lambda , \boldsymbol{r}_{0}} ) {}^{i} \equiv \sum_{j} ( \mathbf{F} \vert _{\lambda , \boldsymbol{r}_{0}} ){}^{i}{}_{j} \boldsymbol{h}^{j} $$

For a given spatial variation $\boldsymbol{h}$, Equation 12 may be interpreted as a generalized directional derivative of an entire field line $\boldsymbol{r} ( \lambda , \boldsymbol{r}_{0} )$ in the direction $\boldsymbol{v} ( \lambda , \boldsymbol{r}_{0} )$. In other words, the Spatial Propagator $\mathbf{F}$ “propagates” every spatial variation $\boldsymbol{h}$ of the reference point $\boldsymbol{r}_{0}$ along the particular field line in 3D space.

*The matrix representation of the Spatial Propagator* $\mathbf{F} ( \lambda , \boldsymbol{r}_{0} )$ *contains all of the geometric information of the local congruence; that is, it carries the local geometric organization of the field lines within a neighborhood* $\Omega _{0} \ll h_{m}^{3}$ *of a particular field-line solution* $\boldsymbol{r} ( \lambda , \boldsymbol{r}_{0} )$. We give a precise meaning to this statement in Section 3.

### Direct Relation Between the Spatial Propagator and the Vector Field

Like the field lines of the congruence, the Spatial Propagator $\mathbf{F} ( \lambda , \boldsymbol{r}_{0} )$ is a function of both the connectivity parameter $\lambda $ and reference point $\boldsymbol{r}_{0}$. In order to calculate the Spatial Propagator, one may integrate *all* field lines, and then construct the difference Equation 10 between any two neighboring field lines, taking care to evaluate the scale length $h_{m}$ for every field line. However, in typical physical systems of interest the governing equations describe the evolution of the vector field (*e.g.* Faraday’s law, MHD induction, *etc.*), and the associated field lines are derived therefrom. Hence, we seek a formulation of the Spatial Propagator directly from the vector field, which allows the simultaneous calculation of the spatial behavior of *all* field lines within the $h_{m}$ neighborhood.

To illustrate, we give a simple, qualitative derivation as follows. The connectivity parameter $\lambda $ and the reference point $\boldsymbol{r}_{0}$ may be considered independent variables in the family of smooth integral curve solutions $\boldsymbol{r} ( \lambda , \boldsymbol{r}_{0} )$ to the system of Equations 2 and 3. From this perspective, the spatial variation with respect to the reference point may be constructed directly from Equation 2 by

13 $$\begin{aligned} \frac{\partial }{\partial \boldsymbol{r}_{0}} \biggl( \frac{\partial \boldsymbol{r} ( \lambda , \boldsymbol{r}_{0} )}{\partial \lambda } \biggr) =& \frac{\partial }{\partial \boldsymbol{r}_{0}} \bigl( \boldsymbol{B} ( \boldsymbol{r} ) \bigr). \end{aligned}$$

Since the vector field $\boldsymbol{B} ( \boldsymbol{r} )$ is assumed to have smooth components, then the family of integral curve solutions $\boldsymbol{r} ( \lambda , \boldsymbol{r}_{0} )$ is smooth in the variables $\lambda $ and $\boldsymbol{r}_{0}$; hence, mixed partial derivatives commute, and Equation 13 may be written

14 $$\begin{aligned} \frac{\partial }{\partial \lambda } \biggl( \frac{\partial \boldsymbol{r} ( \lambda , \boldsymbol{r}_{0} )}{\partial \boldsymbol{r}_{0}} \biggr) =& \biggl( \frac{\partial \boldsymbol{B} ( \boldsymbol{r} )}{\partial \boldsymbol{r}} \bigg\vert _{\boldsymbol{r} = \boldsymbol{r} ( \lambda , \boldsymbol{r}_{0} )} \biggr) \cdot \biggl( \frac{\partial \boldsymbol{r} ( \lambda , \boldsymbol{r}_{0} )}{\partial \boldsymbol{r} _{0}} \biggr). \end{aligned}$$

By Equation 11, Equation 14 is a matrix evolution equation for the (unknown) Spatial Propagator $\mathbf{F} ( \lambda , \boldsymbol{r}_{0} )$. The $\frac{\partial \boldsymbol{B} ( \boldsymbol{r} )}{\partial \boldsymbol{r}}$ term is simply the rate of change of the vector field with respect to the spatial position $\boldsymbol{r} = \boldsymbol{r} ( \lambda , \boldsymbol{r}_{0} )$; represented as a $3 \times 3$ matrix with respect to Cartesian basis,

15 $$\begin{aligned} ( \nabla \boldsymbol{B} \vert _{\boldsymbol{r}} ){}^{i} {}_{j} \equiv & \frac{\partial ( \boldsymbol{B} \vert _{\boldsymbol{r}, t} ){}^{i}}{\partial \boldsymbol{r}^{j}} = \left ( \textstyle\begin{array} {c@{\quad }c@{\quad }c} \frac{\partial B^{x}}{\partial x} & \frac{\partial B^{x}}{\partial y} & \frac{\partial B^{x}}{\partial z} \\ \frac{\partial B^{y}}{\partial x} & \frac{\partial B^{y}}{\partial y} & \frac{\partial B^{y}}{\partial z} \\ \frac{\partial B^{z}}{\partial x} & \frac{\partial B^{z}}{\partial y} & \frac{\partial B^{z}}{\partial z} \end{array}\displaystyle \right ), \end{aligned}$$

where each partial derivative component is evaluated along the field-line component functions, Equation 5.

Substituting Equations 11 and 15 into Equation 14, the Spatial Propagator $\mathbf{F} ( \lambda , \boldsymbol{r}_{0} )$ satisfies the following first-order matrix differential equation, in components with respect to a Cartesian basis $\lbrace \hat{\boldsymbol{e}}_{x}, \hat{\boldsymbol{e}}_{y}, \hat{\boldsymbol{e}}_{z} \rbrace $:

16 $$\begin{aligned} \frac{\partial ( \mathbf{F} \vert _{\lambda , \boldsymbol{r}_{0}} ){}^{i}{}_{j}}{\partial \lambda } =& \sum_{k} ( \nabla \boldsymbol{B} \vert _{\boldsymbol{r}} ){}^{i}{}_{k} ( \mathbf{F} \vert _{\lambda , \boldsymbol{r}_{0}} ){}^{k}{} _{j}, \end{aligned}$$

17 $$\begin{aligned} ( \mathbf{F} \vert _{0 , \boldsymbol{r}_{0}} ){}^{i} {}_{j} =& \delta ^{i}{}_{j}, \end{aligned}$$

where $\delta ^{i}{}_{j}$ is the Kronecker delta.

We remark that by Equation 4 the matrix ODE, Equation 16, is the Lie transport (see, *e.g.*, Kobayashi and Nomizu, 1963, p. 29) of the propagated shift vector $\boldsymbol{v}$ along the vector field $\boldsymbol{B}$,

18 $$\begin{aligned} \mathcal{L}_{\boldsymbol{B}} ( \boldsymbol{v} ) = \mathbf{0} \end{aligned}$$

where the reference shift vector $\boldsymbol{h}$ is a constant vector independent of the coordinates; that is $( \boldsymbol{B} \cdot \nabla ) \boldsymbol{h} = \mathrm{0}$.

In general, Equations 2 and 16, along with their respective initial conditions given by by Equations 3 and 17, are generally referred to as the *equations of variation* (see, *e.g.*, Arnold, 1992, pp. 223 – 225), and they constitute a well-posed problem. Hence, the Spatial Propagator $\mathbf{F} ( \lambda , \boldsymbol{r}_{0} )$ solution exists and is unique for all $\lambda $ and $\boldsymbol{r}_{0}$ (see, *e.g.*, Bernstein, 2018, pp. 1193 – 1195, for existence and uniqueness proof).

*For any reference point* $\boldsymbol{r}_{0}$ *and all* $\lambda $*, the congruence of scale* $h_{m}$ *is a particular solution to the equations of variation consisting of both the integral curve* $\boldsymbol{r} ( \lambda , \boldsymbol{r}_{0} )$ *and corresponding Spatial Propagator* $\mathbf{F} ( \lambda , \mathbf{r}_{0} )$*. The congruence may be interpreted as a local phase-space of scale* $h_{m}$ *for the field-line connectivity consisting of elements* $\boldsymbol{r} ( \lambda , \boldsymbol{r}_{0} )$ *and* $\mathbf{F} ( \lambda , \mathbf{r}_{0} )$*.* With respect to the global Cartesian coordinates, these phase space elements are represented by a three-component vector and $3 \times 3$-component matrix,

19 $$\begin{aligned} \begin{aligned} \boldsymbol{r} ( \lambda , \boldsymbol{r}_{0} ) &= \sum_{i} ( \boldsymbol{r} \vert _{\lambda , \boldsymbol{r}_{0}} ) {}^{i} \hat{\boldsymbol{e}}_{i},\\ \mathbf{F} ( \lambda , \boldsymbol{r}_{0} ) &= \sum _{i,j} ( \mathbf{F} \vert _{\lambda , \boldsymbol{r}_{0}} ) {}^{i}{}_{j} \hat{\boldsymbol{e}}_{i} \otimes \hat{\boldsymbol{e}}^{j}, \end{aligned} \end{aligned}$$

where $\lbrace \hat{\boldsymbol{e}}_{x}, \hat{\boldsymbol{e}}_{y}, \hat{\boldsymbol{e}}_{z} \rbrace $ are the orthonormal basis vectors, and $\lbrace \hat{\boldsymbol{e}}^{x}, \hat{\boldsymbol{e}}^{y}, \hat{\boldsymbol{e}}^{z} \rbrace $ the dual basis (see Appendix B.1).

### General Representations of the Spatial Propagator

There are two fundamental representation categories of the Spatial Propagator $\mathbf{F} ( \lambda , \boldsymbol{r}_{0} )$: the matrix representation with respect to a particular basis set $\hat{\boldsymbol{e}}_{i}$, and the representation with respect to the connectivity parameter $\lambda $.

First, we consider the basis representation of the congruence; that is the field line $\boldsymbol{r} ( \lambda , \boldsymbol{r}_{0} )$ and associated Spatial Propagator $\mathbf{F} ( \lambda , \boldsymbol{r}_{0} )$ are, respectively, a three-component vector and $3 \times 3$ matrix representation *constructed with respect to a particular basis set*. Fundamentally, the matrix representation of the congruence *follows from* the vector field $\boldsymbol{B} ( \boldsymbol{r} )$ and its gradient matrix $\nabla \boldsymbol{B} ( \boldsymbol{r} )$; that is constructing $\boldsymbol{B}$ and $\nabla \boldsymbol{B}$ with respect to Cartesian basis vectors lead to the Cartesian representation of the congruence: Equation 19.

In the Cartesian representation, the basis vectors are independent of the spatial position and hence Equations 11 and 15 find a particularly simple form. However, general curvilinear basis vectors are position dependent (see Appendix B.1); for example, consider the spherical–polar orthonormal^4^ basis vectors,

20 $$\begin{aligned} \begin{aligned} \hat{\boldsymbol{e}}_{r} ( \theta , \phi ) &= \sin \theta \cos \phi \hat{\boldsymbol{e}}_{x} + \sin \theta \sin \phi \hat{ \boldsymbol{e}}_{y} + \cos \theta \hat{\boldsymbol{e}}_{z} \\ \hat{\boldsymbol{e}}_{\theta } ( \theta , \phi ) &= \cos \theta \cos \phi \hat{\boldsymbol{e}}_{x} + \cos \theta \sin \phi \hat{ \boldsymbol{e}}_{y} - \sin \theta \hat{\boldsymbol{e}}_{z} \\ \hat{\boldsymbol{e}}_{\phi } ( \theta , \phi ) &= -\sin \phi \hat{ \boldsymbol{e}}_{x} + \cos \phi \hat{\boldsymbol{e}}_{y}, \end{aligned} \end{aligned}$$

prevalent within solar and space physics. In order to incorporate the spatial position dependence of general curvilinear basis vectors in this formulation, *we impose the condition that the Spatial Propagator* $\mathbf{F} ( \lambda , \boldsymbol{r}_{0} )$ *transform as a tensor under general coordinate transformations*.

The tensor-condition requires the $\nabla \boldsymbol{B} ( \boldsymbol{r} )$ object to be the matrix representation of the covariant differential of the vector field (see, *e.g.*, Kobayashi and Nomizu, 1963, pp. 143 – 144). The components of the matrix representation of the covariant differential of the vector field with respect to general local curvilinear coordinates $q^{i}$ is given by

21 $$\begin{aligned} ( \nabla \boldsymbol{B} \vert _{\boldsymbol{r}} ){}^{i} {}_{j} =& \frac{\partial }{\partial q^{j}} ( \boldsymbol{B} \vert _{\boldsymbol{r}} ){}^{i} + \sum_{k} ( \Gamma \vert _{\boldsymbol{r}} ){}^{i}{}_{jk} ( \boldsymbol{B} \vert _{\boldsymbol{r}} ){}^{k} \end{aligned}$$

(see Appendix B.1 for derivation and discussion). The first term is simply the rate of change of the component functions with respect to the local coordinates. The $( \Gamma \vert _{\boldsymbol{r}} ){}^{i}{}_{jk}$ in the second term are coefficients that account for the spatial position dependence of the basis vectors $\boldsymbol{q}_{i} ( q^{j} )$. Equation 16 with Equation 21, and the initial condition Equation 17, generalizes the field-line deviation to general curvilinear coordinates (see, *e.g.*, Tassev and Sevcheva, 2017, for application in spherical–polar orthonormal coordinates). In Appendices B.2 and B.3 we develop explicit matrix representations of the covariant differential of the vector field $\nabla \boldsymbol{B} ( \boldsymbol{r} )$ with respect to the common spherical–polar bases used by the solar and space-physics community (see, *e.g.*, Tassev and Sevcheva, 2017).

Second, we consider the connectivity parameter representation of the congruence. For a given vector field $\boldsymbol{B} ( \boldsymbol{r} )$, the congruence solution $\boldsymbol{r} ( \lambda , \boldsymbol{r}_{0} )$ and $\mathbf{F} ( \lambda , \boldsymbol{r}_{0} )$ to Equations 2 and 16 with respect to $\lambda $ is said to be given in the *natural representation*. That is the connectivity parameter $\lambda $ has units of arc length per vector field magnitude, and the congruence elements, $\boldsymbol{r} ( \lambda , \boldsymbol{r}_{0} )$ and $\mathbf{F} ( \lambda , \boldsymbol{r}_{0} )$, reflect this functional dependence.

Recall from the end of Section 2.1, the congruence represents an equivalence class under general affine transformations of the connectivity parameter, $\lambda \mapsto \ell ( \lambda ) = f ( \boldsymbol{r} ) \lambda + b$ for smooth, positive-definite functions $f ( \boldsymbol{r} )$ and scalars $b$. Hence, the matrix differential Equations 16 and 17 transform accordingly; with respect to Cartesian coordinates

22 $$\begin{aligned} \frac{\partial ( \mathbf{F} \vert _{\ell , \boldsymbol{r}_{b}} ){}^{i}{}_{j}}{\partial \ell } =& \sum_{k} \biggl( ( \nabla \boldsymbol{X} \vert _{\boldsymbol{r}} ){}^{i}{}_{k} + \frac{ ( \boldsymbol{X} \vert _{\boldsymbol{r}} ){}^{i}}{f ( \boldsymbol{r} )} \frac{\partial f ( \boldsymbol{r} )}{ \partial r^{k}} \biggr) ( \mathbf{F} \vert _{\ell , \boldsymbol{r}_{b}} ){}^{k}{}_{j}, \end{aligned}$$

23 $$\begin{aligned} ( \mathbf{F} \vert _{b, \boldsymbol{r}_{b}} ){}^{i} {}_{j} =& \delta ^{i}{}_{j}. \end{aligned}$$

where $\boldsymbol{X} ( \boldsymbol{r} ) = \boldsymbol{B} ( \boldsymbol{r} )\ / f ( \boldsymbol{r} )$, and the positions are evaluated along $\boldsymbol{r} = \boldsymbol{r} ( \ell , \boldsymbol{r}_{b} )$. Equation 22 with initial condition 23, generalizes the field-line deviation to include affine transformations in the connectivity parameter representations (see, *e.g.*, Tassev and Sevcheva, 2017; Scott, Pontin, and Hornig, 2017, for application).

Explicitly, recall the arc-length representation example, Equations 8 and 9, with $f ( \boldsymbol{r} ) = \vert \boldsymbol{B} ( \boldsymbol{r} ) \vert $, and $\vert \boldsymbol{B} ( \boldsymbol{r} ) \vert \ne 0$. The vector field $\boldsymbol{X} ( \boldsymbol{r} ) = \boldsymbol{b} ( \boldsymbol{r} )$ is well defined and identified with the unit magnetic-field direction. In this example, the arc-length representation of the Spatial Propagator with respect to Cartesian coordinates satisfies

24 $$\begin{aligned} \frac{\partial ( \mathbf{F} \vert _{\ell , \boldsymbol{r}_{0}} ){}^{i}{}_{j}}{\partial \ell } =& \sum_{k} \biggl( ( \nabla \boldsymbol{b} \vert _{\boldsymbol{r}} ){}^{i}{}_{k} + \frac{( \boldsymbol{b} \vert _{\boldsymbol{r}} ){}^{i}}{\vert \boldsymbol{B} ( \boldsymbol{r} ) \vert } \frac{\partial \vert \boldsymbol{B} ( \boldsymbol{r} ) \vert }{\partial r^{k}} \biggr) ( \mathbf{F} \vert _{\ell , \boldsymbol{r}_{0}} ){}^{k} {}_{j}, \end{aligned}$$

25 $$\begin{aligned} ( \mathbf{F} \vert _{0 , \boldsymbol{r}_{0}} ){}^{i} {}_{j} =& \delta ^{i}{}_{j}. \end{aligned}$$

where the positions are evaluated along the arc-length representation of the field line, $\boldsymbol{r} = \boldsymbol{r} ( \ell , \boldsymbol{r}_{0} )$.

In the application to solar magnetic fields, constructing the congruence solution from data (*e.g.* *Solar Dynamics Observatory*/*Atmospheric Imaging Assembly* 171 Å images) requires the solutions to Equations 8 and 24, with initial conditions 7 and 25, respectively. From this perspective, we have decoupled the magnetic-field strength estimation from the field-line trajectory estimation in the construction of the Spatial Propagator.

## Geometric Deformation of a Congruence

Geometry describes the measurable lengths and angles associated with the system configuration. The value of the congruence formalism is that all of the geometric behavior of a local bundle of field lines is contained within a single integration of Equations 2 and 16, along with their respective initial conditions 3 and 17. The field line $\boldsymbol{r} ( \lambda , \boldsymbol{r}_{0} )$ and corresponding Spatial Propagator $\mathbf{F} ( \lambda , \boldsymbol{r}_{0} )$ implicitly incorporate all geometric information of the local bundle, which follows naturally from the vector magnetic-field structure: $\boldsymbol{B}$ and $\nabla \boldsymbol{B}$. The generic geometric configuration of the congruence may be described by a combination of dilation (Section 3.1), stretch, rotation (Section 3.2), and gradients in the connectivity structure (Section 3.3).

We recall that by assumption the vector field components are smooth functions. Hence, by standard theorems of existence, uniqueness, and extension for ordinary differential equations (see, *e.g.*, Hirsch and Smale, 1974; Arnold, 1992; Taylor, 1996), each field-line reference point $\boldsymbol{r}_{0} \in \Omega _{0}$ is mapped smoothly and uniquely to the point $\boldsymbol{r} = \boldsymbol{r} \left ( \lambda , \boldsymbol{r}_{0} \right ) \in \Omega _{\lambda }$ (see Figure 1); that is the neighborhood volume $\Omega _{0}$ is mapped, smoothly and uniquely, to the neighborhood volume $\Omega _{\lambda }$. Hence, *the geometric deformation of the local congruence is reflected in the deformation of the initial volume* $\Omega _{0}$ *by propagation along the congruence into* $\Omega _{\lambda }$.

### Congruence Dilation and the Determinant of the Spatial Propagator

The dilation of a congruence is the compression/expansion of the constituent field lines, and it is completely described by the determinant of the $3 \times 3$, non-singular (invertible) matrix representation of the Spatial Propagator $\mathbf{F} ( \lambda , \boldsymbol{r}_{0} )$. This type of geometric deformation is quantified by the dilation of a volume $\Omega _{\lambda } \subseteq \mathbb{R}^{3}$ propagated through each $\lambda $ along the congruence. In this section, we make explicit the functional dependence of the deformed volume on both the connectivity parameter $\lambda $ and the reference point $\boldsymbol{r}_{0}$, by denoting $\Omega _{\lambda } = \Omega ( \lambda , \boldsymbol{r}_{0} )$.

The signed differential volume element in Cartesian coordinates is constructed from the coordinate differentials $\mathrm{d}x^{i} \,\hat{\boldsymbol{e}}_{i}$,

26 $$ {\mathrm{d}} ^{3} \boldsymbol{x} = ( {\mathrm{d}} z\, \hat{\boldsymbol{e}}_{z} ) \cdot \bigl( ( {\mathrm{d}} x \, \hat{ \boldsymbol{e}}_{x} ) \times ( {\mathrm{d}} y \hat{ \boldsymbol{e}}_{y} ) \bigr) = \hat{\boldsymbol{e}}_{z} \cdot ( \hat{\boldsymbol{e}}_{x} \times \hat{\boldsymbol{e}}_{y} )\, {\mathrm{d}} x\, {\mathrm{d}} y\, {\mathrm{d}} z. $$

We may choose differential-reference-shift vectors to coincide with the Cartesian coordinate differentials ${\mathrm{d}} \boldsymbol{h}^{i} \equiv {\mathrm{d}} x^{i} \hat{\boldsymbol{e}}_{i}$, and hence write the local signed differential volume element at the reference point ${\mathrm{d}} \Omega ( 0, \boldsymbol{r}_{0} ) = {\mathrm{d}} ^{3} \boldsymbol{x}$.

**Figure 3:**
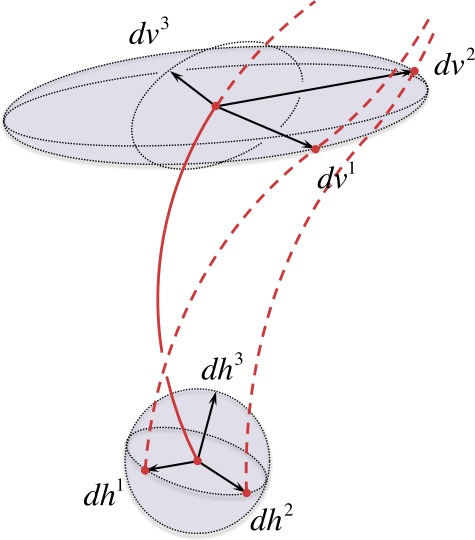
Illustration of the volumetric propagation under the action of the Spatial Propagator. Each ${\mathrm{d}} \boldsymbol{h}^{i}$ is propagated to ${\mathrm{d}} \boldsymbol{v}^{i}$. The propagated vectors illustrated are linearly independent, and otherwise completely general.

Each differential-reference-shift vector ${\mathrm{d}} \boldsymbol{h} ^{i}$ may be propagated along the congruence under the action of $\mathbf{F} ( \lambda , \boldsymbol{r}_{0} )$ (see Figure 3), such that

27 $$\begin{aligned} {\mathrm{d}} \boldsymbol{v}^{i} ( \lambda , \boldsymbol{r}_{0} ) =& \mathbf{F} ( \lambda , \boldsymbol{r}_{0} ) \cdot {\mathrm{d}} \boldsymbol{h}^{i} = {\mathrm{d}} x^{i} \,\mathbf{F} ( \lambda , \boldsymbol{r}_{0} ) \cdot \hat{\boldsymbol{e}} _{i}, \end{aligned}$$

where for each $i = x, y, z$, the $\mathbf{F} ( \lambda , \boldsymbol{r}_{0} ) \cdot \hat{\boldsymbol{e}}_{i}$ is a vector; expanded with respect to Cartesian basis (see Appendix C.1),

28 $$\begin{aligned} \mathbf{F} ( \lambda , \boldsymbol{r}_{0} ) \cdot \hat{\boldsymbol{e}}_{i} =& ( \mathbf{F} \vert _{\lambda , \boldsymbol{r}_{0}} ){}^{x}{}_{i} \hat{\boldsymbol{e}}_{x} + ( \mathbf{F} \vert _{\lambda , \boldsymbol{r}_{0}} ){}^{y}{}_{i} \hat{ \boldsymbol{e}}_{y} + ( \mathbf{F} \vert _{\lambda , \boldsymbol{r}_{0}} ){}^{z}{}_{i} \hat{\boldsymbol{e}}_{z}. \end{aligned}$$

Hence, the local signed differential volume element propagated along the congruence is completely determined by the action of the Spatial Propagator on the basis vectors:

29 $$\begin{aligned} {\mathrm{d}} \Omega ( \lambda , \boldsymbol{r}_{0} ) =& {\mathrm{d}} \boldsymbol{v}^{z} ( \lambda , \boldsymbol{r}_{0} ) \cdot \bigl( {\mathrm{d}} \boldsymbol{v}^{x} ( \lambda , \boldsymbol{r}_{0} ) \times {\mathrm{d}} \boldsymbol{v}^{y} ( \lambda , \boldsymbol{r}_{0} ) \bigr) \\ =& \bigl( \mathbf{F} ( \lambda , \boldsymbol{r}_{0} ) \cdot \hat{ \boldsymbol{e}}_{z} \bigr) \cdot \bigl( \bigl( \mathbf{F} ( \lambda , \boldsymbol{r}_{0} ) \cdot \hat{\boldsymbol{e}}_{x} \bigr) \times \bigl( \mathbf{F} ( \lambda , \boldsymbol{r}_{0} ) \cdot \hat{ \boldsymbol{e}}_{y} \bigr) \bigr) \,{\mathrm{d}} x\, {\mathrm{d}} y \,{ \mathrm{d}} z. \end{aligned}$$

By a standard result from vector calculus in three dimensions, the ratio of propagated volume element to initial volume element is given by the determinant of the matrix representation of $\mathbf{F} ( \lambda , \boldsymbol{r}_{0} )$:

30 $$\begin{aligned} \frac{ {\mathrm{d}} \Omega ( \lambda , \boldsymbol{r}_{0} )}{ {\mathrm{d}} \Omega ( 0 , \boldsymbol{r}_{0} )} =& \frac{ ( \mathbf{F} ( \lambda , \boldsymbol{r}_{0} ) \cdot \hat{\boldsymbol{e}}_{z} ) \cdot ( ( \mathbf{F} ( \lambda , \boldsymbol{r}_{0} ) \cdot \hat{\boldsymbol{e}}_{x} ) \times ( \mathbf{F} ( \lambda , \boldsymbol{r}_{0} ) \cdot \hat{\boldsymbol{e}}_{y} ) )}{\hat{\boldsymbol{e}}_{z} \cdot ( \hat{\boldsymbol{e}}_{x} \times \hat{\boldsymbol{e}}_{y} )}, \\ =& \operatorname{det} \mathbf{F} ( \lambda , \boldsymbol{r}_{0} ). \end{aligned}$$

In Appendix C.1, we calculate Equation 30 explicitly for a Cartesian basis. Furthermore, identity 30 is valid for arbitrary orthonormal basis sets (see, *e.g.*, Nickerson, Spencer, and Steenrod, 1959, pp. 95 – 97, for a standard treatment). From a geometric perspective, the determinant of the $3 \times 3$ matrix representation of $\mathbf{F} ( \lambda , \boldsymbol{r}_{0} )$ acts as the Jacobian in a change of basis for the signed volume element propagated along the congruence (see Figure 3).

Since the differential-reference-shift vectors ${\mathrm{d}} \boldsymbol{h}^{i}$ are mapped under the action of the Spatial Propagator into the propagated differential vectors ${\mathrm{d}} \boldsymbol{v} ^{i} ( \lambda , \boldsymbol{r}_{0} )$, the signed differential volume elements ${\mathrm{d}} \Omega ( 0 , \boldsymbol{r}_{0} )$ and ${\mathrm{d}} \Omega ( \lambda , \boldsymbol{r}_{0} )$ are centered on the respective points $\boldsymbol{r}_{0} = \boldsymbol{r} ( 0 , \boldsymbol{r}_{0} )$ and $\boldsymbol{r} = \boldsymbol{r} ( \lambda , \boldsymbol{r}_{0} )$. Hence, 3D integration with respect to the volume measure ${\mathrm{d}} \Omega ( \lambda , \boldsymbol{r}_{0} )$ generates a total volume $\Omega ( \lambda , \boldsymbol{r}_{0} )$, deformed with respect to the initial shape $\Omega ( 0, \boldsymbol{r}_{0} )$ and centered on the field line $\boldsymbol{r} ( \lambda , \boldsymbol{r}_{0} )$ at each value of $\lambda $. Moreover, by Equation 30 the deformed total volume at every $\lambda $ may be cast as an integral with respect to the reference volume measure ${\mathrm{d}} \Omega ( 0 , \boldsymbol{r}_{0} )$ at the reference point $\boldsymbol{r}_{0}$,

31 $$ \Omega ( \lambda , \boldsymbol{r}_{0} ) = \int _{\Omega ( \lambda , \boldsymbol{r}_{0} )} {\mathrm{d}} \Omega ( \lambda , \boldsymbol{r}_{0} ) = \int _{\Omega ( 0, \boldsymbol{r}_{0} )} \operatorname{det} \mathbf{F} ( \lambda , \boldsymbol{r}_{0} ) \,{\mathrm{d}} \Omega ( 0, \boldsymbol{r}_{0} ). $$

The dilation of the total volume $\Omega ( \lambda , \boldsymbol{r}_{0} )$ by propagation along the congruence is therefore completely determined by the determinant of the Spatial Propagator $\operatorname{det} \mathbf{F} ( \lambda , \boldsymbol{r} _{0} )$ at each $\lambda $. Geometrically, the total volume $\Omega ( \lambda , \boldsymbol{r}_{0} )$ decreases (volumetric compression) for $0 < \operatorname{det} \mathbf{F} ( \lambda , \boldsymbol{r}_{0} ) < 1$, remains constant (isochoric propagation) for $\operatorname{det} \mathbf{F} ( \lambda , \boldsymbol{r}_{0} ) = 1$, or increases (volumetric expansion) for $\operatorname{det} \mathbf{F} ( \lambda , \boldsymbol{r}_{0} ) > 1$, respectively.

We remark, $\operatorname{det}\mathbf{F} ( \lambda , \boldsymbol{r}_{0} ) < 0$ corresponds to a mapping from a proper right-handed basis set, to a left-handed basis set. Such an $\mathbf{F} ( \lambda , \boldsymbol{r}_{0} )$ is discontinuous and requires singularities in the vector field $\boldsymbol{B}$, which we do not consider in this work.

Moreover, using Equation 31, we can quantify the rate of volumetric dilation by propagation along the congruence,

32 $$\begin{aligned} \frac{\partial \Omega ( \lambda , \boldsymbol{r}_{0} )}{ \partial \lambda } =& \int _{\Omega ( 0, \boldsymbol{r}_{0} )} \frac{\partial \operatorname{det} \mathbf{F} ( \lambda , \boldsymbol{r}_{0} )}{\partial \lambda }\, {\mathrm{d}} \Omega ( 0, \boldsymbol{r}_{0} ), \\ =& \int _{\Omega ( 0, \boldsymbol{r}_{0} )} {\operatorname{Tr}} \bigl( \nabla \boldsymbol{B} ( \boldsymbol{r} ) \bigr) \operatorname{det} \mathbf{F} ( \lambda , \boldsymbol{r}_{0} )\, {\mathrm{d}} \Omega ( 0, \boldsymbol{r}_{0} ) \end{aligned}$$

where we have made use of the identity

33 $$ \frac{\partial \operatorname{det} \mathbf{F} ( \lambda , \boldsymbol{r}_{0} )}{\partial \lambda } = \operatorname{Tr} \bigl( \ \nabla \boldsymbol{B} ( \boldsymbol{r} ) \bigr) \operatorname{det} \mathbf{F} ( \lambda , \boldsymbol{r}_{0} ), $$

(see Appendix C.2 for derivation).

In Equations 32 and 33, the $\operatorname{Tr} ( \nabla \boldsymbol{B} ( \boldsymbol{r} ) \ )$ term is evaluated at each point $\boldsymbol{r} = \boldsymbol{r} ( \lambda , \boldsymbol{r}_{0} )$. In particular, every physical magnetic vector field is everywhere divergence-free: $\operatorname{Tr} ( \nabla \boldsymbol{B} ( \boldsymbol{r} ) ) = \nabla \cdot \boldsymbol{B} ( \boldsymbol{r} ) = 0$ for all $\boldsymbol{r} \in M$. Hence, by Equations 17 and 33, for all finite $\lambda $,

34 $$ {\operatorname{det}} \mathbf{F} ( \lambda , \boldsymbol{r}_{0} ) = \operatorname{det} \mathbf{F} ( 0, \boldsymbol{r}_{0} ) = \operatorname{det} \mathbf{I} = 1. $$

This implies that the total volume is conserved under propagation along a congruence; that is $\Omega ( \lambda , \boldsymbol{r}_{0} ) = \Omega ( 0, \boldsymbol{r}_{0} )$ is constant regardless of deformation. Hence, *we identify* $\operatorname{det} \mathbf{F} ( \lambda , \boldsymbol{r}_{0} ) = 1$ *for all* $\lambda $ *and every* $\boldsymbol{r}_{0}$ *as the simplest topological invariant of a congruence generated by a smooth magnetic vector field. This true topological invariant reflects the divergence-free condition.*

### Congruence Stretch and Rotation and the Singular Value Decomposition of the Spatial Propagator

**Figure 4:**
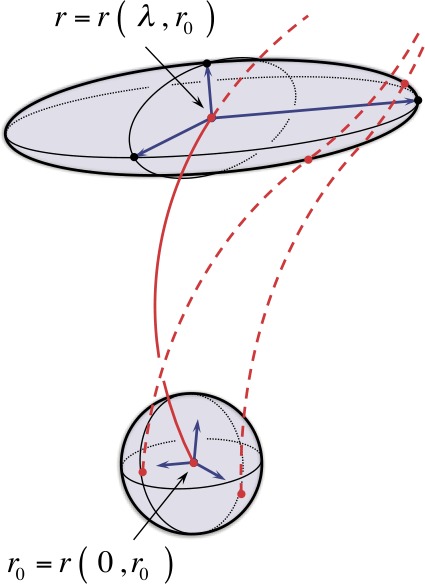
Illustration of an anisotropic stretch and rigid-body rotation of an orthogonal frame centered about the central field-line trajectory $\boldsymbol{r} ( \lambda , \boldsymbol{r}_{0} )$ within the propagated volume. In contrast to Figure 3, a specific orthogonal frame ${\hat{\boldsymbol{r}}}_{\alpha }$ is shown centered on $\boldsymbol{r} ( 0, \boldsymbol{r}_{0} )$. It is rotated along the central field line into the specific orthogonal frame ${\hat{\boldsymbol{l}}}_{\alpha }$ centered at $\boldsymbol{r} ( \lambda , \boldsymbol{r}_{0} )$ while the vector lengths are simultaneously scaled by the corresponding $\sigma _{\alpha }$ as defined in Section 3.2.

The anisotropic stretch and rigid-body rotation of a congruence are equivalent to the kinematic description of the propagated volume undergoing similar deformation; that is, the $\lambda $-evolution of the propagated vectors $\boldsymbol{v} ( \lambda , \boldsymbol{r}_{0} )$ with respect to a local volume-centered orthogonal reference frame. The congruence stretch and rotation is described by a deformation scaled along mutually orthogonal, body-centered axes of the propagated volume. This basic geometric picture is illustrated in Figure 4.

We seek a geometric description in which the orthogonality of the reference frame is preserved under rotation and the lengths of the direction vectors are scaled. This description initially suggests the $\lambda $-evolution of the eigen-decomposition of the Spatial Propagator matrix $\mathbf{F}$, in which the eigenvalues and eigenvectors play the role of scale factors and basis directions. The eigenvalues for a general, non-singular, $3 \times 3$ matrix $\mathbf{F}$ are determined by a cubic polynomial with real coefficients, the roots of which may be real or complex-valued depending on the sign of the classical cubic discriminant. In the case of non-negative cubic discriminant, the eigenvalues are all real (perhaps with multiplicity $> 1$), the geometric interpretation is scaling along linearly independent eigenvectors (since $\mathbf{F}$ is a non-singular matrix). However, *mutual orthogonality of the eigenvectors follows only in the special case that the Spatial Propagator matrix is symmetric*, $\mathbf{F} = \mathbf{F}^{T}$ (where $\mathbf{F}^{T}$ denotes the matrix transpose). Moreover, a negative cubic discriminant corresponds to complex-valued eigenvalues and eigenvectors, the geometric interpretation of which is not a stretch and rotation of the propagated volume. Hence, the eigen-decomposition of the Spatial Propagator $\mathbf{F}$ does not provide the correct stretch and rotation description.

To determine the correct description, consider that a general stretch is described by a *change in the length* of propagated vectors $\boldsymbol{v} \left ( \lambda , \boldsymbol{r}_{0} \right )$ within the propagated volume. The length of a vector is described by the norm

35 $$\begin{aligned} \bigl\vert \boldsymbol{v} ( \lambda , \boldsymbol{r}_{0} ) \bigr\vert =& \bigl( \boldsymbol{v} ( \lambda , \boldsymbol{r}_{0} ) \cdot \boldsymbol{v} ( \lambda , \boldsymbol{r}_{0} ) \bigr) {}^{1/2} \\ =& \bigl( \boldsymbol{h} \cdot \mathbf{F}^{T} ( \lambda , \boldsymbol{r}_{0} ) \cdot \mathbf{F} ( \lambda , \boldsymbol{r}_{0} ) \cdot \boldsymbol{h} \bigr){}^{1/2}. \end{aligned}$$

Equation 35 is a real-valued function of $\lambda $ that involves the symmetric matrix $\mathbf{F}^{T} \cdot \mathbf{F}$, as opposed to the Spatial Propagator $\mathbf{F}$ alone. By a standard theorem of linear algebra (see, *e.g.*, Halmos, 1958, Section 79), the eigenvalues of the symmetric matrix $\mathbf{F}^{T} \cdot \mathbf{F}$ are all real (possibly with multiplicity $> 1$), and the eigenvectors are everywhere mutually orthogonal. More generally, the symmetric matrices $\mathbf{F}^{T} \cdot \mathbf{F}$ and $\mathbf{F} \cdot \mathbf{F} ^{T}$ have identical eigenvalues, as well as orthogonal, albeit different, eigenvector bases. Hence, for a general, non-singular Spatial Propagator $\mathbf{F}$, the real eigenvalues and real orthogonal eigenvectors of the symmetric matrices $\mathbf{F} \cdot \mathbf{F} ^{T}$, respectively $\mathbf{F}^{T} \cdot \mathbf{F}$, may be used to infer indirectly the geometric stretch and rotation interpretation of $\mathbf{F}$.

Formally, for each $\boldsymbol{r}_{0}$ and all $\lambda $, there exists a factorization of $\mathbf{F} ( \lambda , \boldsymbol{r}_{0} )$, called the *singular value decomposition* (SVD), given by

36 $$\begin{aligned} \mathbf{F} ( \lambda , \boldsymbol{r}_{0} ) = \mathbf{R} _{l} ( \lambda , \boldsymbol{r}_{0} ) \cdot \mathbf{P} ( \lambda , \boldsymbol{r}_{0} ) \cdot \mathbf{R}_{r} ( \lambda , \boldsymbol{r}_{0} ), \end{aligned}$$

where $\mathbf{P} ( \lambda , \boldsymbol{r}_{0} )$ is a $3 \times 3$ diagonal matrix, and the $\mathbf{R}_{l} ( \lambda , \boldsymbol{r}_{0} )$ and $\mathbf{R}_{r} ( \lambda , \boldsymbol{r}_{0} )$ are $3 \times 3$ orthogonal matrices (*e.g.* Bernstein, 2018, pp. 555 – 558). The diagonal entries of the matrix $\mathbf{P} ( \lambda , \boldsymbol{r}_{0} )$ are non-negative functions $\sigma _{\alpha } ( \lambda , \boldsymbol{r}_{0} )$ for $\alpha = 1, 2, 3$, given by

37 $$\begin{aligned} \sigma _{\alpha } ( \lambda , \boldsymbol{r}_{0} ) = \sqrt{q _{\alpha } ( \lambda , \boldsymbol{r}_{0} )}, \end{aligned}$$

where $q_{\alpha } ( \lambda , \boldsymbol{r}_{0} )$ are the eigenvalues of the matrices $\mathbf{F}^{T} \cdot \mathbf{F}$ and $\mathbf{F} \cdot \mathbf{F}^{T}$. The columns of $\mathbf{R}_{l} ( \lambda , \boldsymbol{r}_{0} )$, denoted by $\hat{\boldsymbol{l}}_{\alpha } ( \lambda , \boldsymbol{r}_{0} )$, are the mutually orthogonal eigenvectors of the symmetric matrix $\mathbf{F} \cdot \mathbf{F}^{T}$; respectively, the columns of $\mathbf{R}_{r} ( \lambda , \boldsymbol{r}_{0} )$, denoted by $\hat{\boldsymbol{r}}_{\alpha } ( \lambda , \boldsymbol{r}_{0} )$, are the mutually orthogonal eigenvectors of the symmetric matrix $\mathbf{F}^{T} \cdot \mathbf{F}$.

For each reference point $\boldsymbol{r}_{0}$ and fixed $\lambda $, the set of values $\sigma _{\alpha }$ for $\alpha = 1, 2, 3$ are called the *singular values* of $\mathbf{F} ( \lambda , \boldsymbol{r} _{0} )$, and the corresponding set of vectors $\hat{{\boldsymbol{l}}}_{\alpha }$ and ${\hat{\boldsymbol{r}}}_{\alpha }$ are called the *singular vectors* of $\mathbf{F} ( \lambda , \boldsymbol{r}_{0} )$. The anisotropic stretch and rigid-body rotation of the propagated volume, equivalently the congruence geometry, is completely determined by the singular values and singular vectors of the Spatial Propagator $\mathbf{F} ( \lambda , \boldsymbol{r}_{0} )$. Geometrically, an orthogonal frame ${\hat{\boldsymbol{r}}} _{\alpha }$ in the reference volume $\Omega _{0}$ is rotated into the orthogonal frame ${\hat{\boldsymbol{l}}}_{\alpha }$ in the propagated volume $\Omega _{\lambda }$ while the vector lengths are simultaneously scaled by the corresponding $\sigma _{\alpha }$, as shown in Figure 4.

We may decompose this simultaneous stretch and rigid-body rotation effect through a *polar decomposition* of the Spatial Propagator. Since $\mathbf{R}_{l} ( \lambda , \boldsymbol{r}_{0} )$ is an orthogonal matrix ($\mathbf{R}^{-1} = \mathbf{R}^{T}$, where superscript “$T$” denotes the matrix transpose) for each $\boldsymbol{r}_{0}$ and all $\lambda $, then $\mathbf{R}_{l}^{T} ( \lambda , \boldsymbol{r}_{0} ) \cdot \mathbf{R}_{l} ( \lambda , \boldsymbol{r}_{0} ) = \mathbf{I}$, where $\mathbf{I}$ is the identity. Hence, we may rewrite Equation 36 as

38 $$\begin{aligned} \mathbf{F} ( \lambda , \boldsymbol{r}_{0} ) =& \mathbf{R} _{l} ( \lambda , \boldsymbol{r}_{0} ) \cdot \mathbf{P} ( \lambda , \boldsymbol{r}_{0} ) \cdot \mathbf{I} \cdot \mathbf{R}_{r} ( \lambda , \boldsymbol{r}_{0} ) \\ =& \mathbf{R}_{l} ( \lambda , \boldsymbol{r}_{0} ) \cdot \mathbf{P} ( \lambda , \boldsymbol{r}_{0} ) \cdot \bigl( \ \mathbf{R}_{l}^{T} ( \lambda , \boldsymbol{r}_{0} ) \cdot \mathbf{R}_{l} ( \lambda , \boldsymbol{r}_{0} ) \ \bigr) \cdot \mathbf{R}_{r} ( \lambda , \boldsymbol{r}_{0} ) \\ =& \bigl( \mathbf{R}_{l} ( \lambda , \boldsymbol{r}_{0} ) \cdot \mathbf{P} ( \lambda , \boldsymbol{r}_{0} ) \cdot \mathbf{R}_{l}^{T} ( \lambda , \boldsymbol{r}_{0} ) \ \bigr) \cdot \bigl( \mathbf{R}_{l} ( \lambda , \boldsymbol{r}_{0} ) \cdot \mathbf{R}_{r} ( \lambda , \boldsymbol{r}_{0} ) \bigr) \\ =& \mathbf{V} ( \lambda , \boldsymbol{r}_{0} ) \cdot \mathbf{R} ( \lambda , \boldsymbol{r}_{0} ). \end{aligned}$$

By the exact same arguments, making use of the orthogonality of $\mathbf{R}_{r} ( \lambda , \boldsymbol{r}_{0} )$, we find an equivalent rewrite of Equation 36 as

39 $$\begin{aligned} \mathbf{F} ( \lambda , \boldsymbol{r}_{0} ) =& \mathbf{R} ( \lambda , \boldsymbol{r}_{0} ) \cdot \mathbf{U} ( \lambda , \boldsymbol{r}_{0} ). \end{aligned}$$

The equivalent decompositions in Equations 38 and 39 are referred to as the *left-polar decomposition* and *right-polar decomposition*, respectively, of the Spatial Propagator (see, *e.g.*, Halmos, 1958, Section 83). Unlike the general Spatial Propagator $\mathbf{F} ( \lambda , \boldsymbol{r}_{0} )$ itself, the matrices $\mathbf{V} ( \lambda , \boldsymbol{r}_{0} )$ and $\mathbf{U} ( \lambda , \boldsymbol{r}_{0} )$, denoted, respectively, the *left-stretch* and *right-stretch*, are symmetric, positive definite, $3 \times 3$ matrices defined by

40 $$\begin{aligned} \begin{aligned} \mathbf{V} ( \lambda , \boldsymbol{r}_{0} ) &\equiv \mathbf{R}_{l} ( \lambda , \boldsymbol{r}_{0} ) \cdot \mathbf{P} ( \lambda , \boldsymbol{r}_{0} ) \cdot \mathbf{R}_{l}^{T} ( \lambda , \boldsymbol{r}_{0} ), \\ \mathbf{U} ( \lambda , \boldsymbol{r}_{0} ) &\equiv \mathbf{R}_{r}^{T} ( \lambda , \boldsymbol{r}_{0} ) \cdot \mathbf{P} ( \lambda , \boldsymbol{r}_{0} ) \cdot \mathbf{R}_{r} ( \lambda , \boldsymbol{r}_{0} ). \end{aligned} \end{aligned}$$

Moreover, the matrix $\mathbf{R} ( \lambda , \boldsymbol{r}_{0} )$ is a $3 \times 3$, orthogonal matrix defined by

41 $$\begin{aligned} \mathbf{R} ( \lambda , \boldsymbol{r}_{0} ) \equiv & \mathbf{R}_{l} ( \lambda , \boldsymbol{r}_{0} ) \cdot \mathbf{R}_{r} ( \lambda , \boldsymbol{r}_{0} ). \end{aligned}$$

The left-stretch $\mathbf{V} ( \lambda , \boldsymbol{r}_{0} )$, right-stretch $\mathbf{U} ( \lambda , \boldsymbol{r} _{0} )$, and rotation $\mathbf{R} ( \lambda , \boldsymbol{r}_{0} )$ matrices all naturally inherit their (global) coordinate representation from the matrix representation of the Spatial Propagator $\mathbf{F} ( \lambda , \boldsymbol{r}_{0} )$.

Equation 40 represent two coordinate rotations by the orthogonal matrices $\mathbf{R}_{l} ( \lambda , \boldsymbol{r} _{0} )$ and $\mathbf{R}_{r} ( \lambda , \boldsymbol{r}_{0} )$ that diagonalize, respectively, the $\mathbf{V} ( \lambda , \boldsymbol{r}_{0} )$ and $\mathbf{U} ( \lambda , \boldsymbol{r}_{0} )$ matrices. The anisotropic stretch and rigid-body rotation geometric interpretation, however, becomes explicit by application of the standard spectral decomposition theorem of linear algebra (see, *e.g.*, Halmos, 1958, Section 79). Since the matrices $\mathbf{V} ( \lambda , \boldsymbol{r}_{0} )$ and $\mathbf{U} ( \lambda , \boldsymbol{r}_{0} )$ are symmetric, there exist orthogonal eigen-bases ${\hat{\boldsymbol{l}}}_{\alpha } ( \lambda , \boldsymbol{r}_{0} )$ and ${\hat{\boldsymbol{r}}} _{\alpha } ( \lambda , \boldsymbol{r}_{0} )$, with respect to which the matrix representations are

42 $$\begin{aligned} \begin{aligned} \mathbf{V} ( \lambda , \boldsymbol{r}_{0} ) &= \sum_{ \alpha } \sigma _{\alpha } ( \lambda , \boldsymbol{r}_{0} ) \hat{\boldsymbol{l}}_{\alpha } ( \lambda , \boldsymbol{r}_{0} ) \otimes \hat{\boldsymbol{l}}_{\alpha } ( \lambda , \boldsymbol{r}_{0} ), \\ \mathbf{U} ( \lambda , \boldsymbol{r}_{0} ) &= \sum _{ \alpha } \sigma _{\alpha } ( \lambda , \boldsymbol{r}_{0} ) \hat{\boldsymbol{r}}_{\alpha } ( \lambda , \boldsymbol{r}_{0} ) \otimes \hat{\boldsymbol{r}}_{\alpha } ( \lambda , \boldsymbol{r}_{0} ). \end{aligned} \end{aligned}$$

By Equations 40 and 41, the eigen-bases ${\hat{\boldsymbol{l}}}_{\alpha } ( \lambda , \boldsymbol{r}_{0} )$ and ${\hat{\boldsymbol{r}}}_{\alpha } ( \lambda , \boldsymbol{r}_{0} )$, of the left-stretch $\mathbf{V} ( \lambda , \boldsymbol{r}_{0} )$, respectively right-stretch $\mathbf{U} ( \lambda , \boldsymbol{r}_{0} )$, matrices are the singular vectors of the Spatial Propagator $\mathbf{F} ( \lambda , \boldsymbol{r}_{0} )$.

Moreover, the singular vectors ${\hat{\boldsymbol{l}}}_{\alpha } ( \lambda , \boldsymbol{r}_{0} )$ and ${\hat{\boldsymbol{r}}}_{\alpha } ( \lambda , \boldsymbol{r}_{0} )$ are related via the rotation matrix $\mathbf{R} ( \lambda , \boldsymbol{r}_{0} )$: Equation 41. By Equations 38 and 39, the right- and left-polar decompositions are equal, and hence, $\mathbf{V} = \mathbf{R} \cdot \mathbf{U} \cdot \mathbf{R}^{-1}$. Moreover, using the eigen-basis representations of Equation 42, and the orthogonality of the rotation matrix $\mathbf{R}^{-1} = \mathbf{R}^{T}$, we find

$$\begin{aligned} \sum_{\alpha } \sigma _{\alpha } { \hat{\boldsymbol{l}}}_{\alpha } \otimes {\hat{\boldsymbol{l}}}_{\alpha } =& \mathbf{R} \cdot \biggl( \sum_{\alpha } \sigma _{\alpha } {\hat{\boldsymbol{r}}}_{\alpha } \otimes { \hat{\boldsymbol{r}}}_{\alpha } \biggr) \cdot \mathbf{R}^{T} \\ =& \sum_{\alpha } \sigma _{\alpha } ( \mathbf{R} \cdot {\hat{\boldsymbol{r}}}_{\alpha } ) \otimes \bigl( { \hat{\boldsymbol{r}}}_{\alpha } \cdot \mathbf{R}^{T} \bigr). \end{aligned}$$

Hence, matching terms

43 $$\begin{aligned} {\hat{\boldsymbol{l}}}_{\alpha } ( \lambda , \boldsymbol{r}_{0} ) =& \mathbf{R} ( \lambda , \boldsymbol{r}_{0} ) \cdot {\hat{\boldsymbol{r}}}_{\alpha } ( \lambda , \boldsymbol{r}_{0} ) = {\hat{\boldsymbol{r}}}_{\alpha } ( \lambda , \boldsymbol{r}_{0} ) \cdot \mathbf{R}^{T} ( \lambda , \boldsymbol{r}_{0} ). \end{aligned}$$

Furthermore, inverting Equation 43 yields,

44 $$\begin{aligned} {\hat{\boldsymbol{r}}}_{\alpha } ( \lambda , \boldsymbol{r}_{0} ) =& {\hat{\boldsymbol{l}}}_{\alpha } ( \lambda , \boldsymbol{r}_{0} ) \cdot \mathbf{R} ( \lambda , \boldsymbol{r}_{0} ) = \mathbf{R}^{T} ( \lambda , \boldsymbol{r}_{0} ) \cdot {\hat{\boldsymbol{l}}}_{\alpha } ( \lambda , \boldsymbol{r}_{0} ). \end{aligned}$$

Using Equations 42 and 44 in the left-polar decomposition Equation 38, the Spatial Propagator $\mathbf{F} ( \lambda , \boldsymbol{r}_{0} )$ matrix may be represented with respect to the singular vector basis ${\hat{\boldsymbol{l}}}_{\alpha } ( \lambda , \boldsymbol{r}_{0} )$ and $\hat{\boldsymbol{r}} _{\alpha } ( \lambda , \boldsymbol{r}_{0} )$,

45 $$\begin{aligned} \mathbf{F} ( \lambda , \boldsymbol{r}_{0} ) =& \sum _{ \alpha } \sigma _{\alpha } ( \lambda , \boldsymbol{r}_{0} ) {\hat{\boldsymbol{l}}}_{\alpha } ( \lambda , \boldsymbol{r}_{0} ) \otimes {\hat{\boldsymbol{r}}}_{\alpha } ( \lambda , \boldsymbol{r}_{0} ). \end{aligned}$$

**Figure 5:**
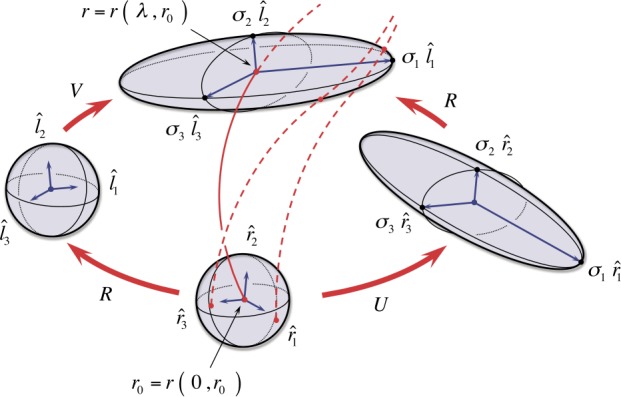
Illustration of the polar decomposition of the Spatial Propagator. The right-polar decomposition, $\mathbf{F} = \mathbf{R} \cdot \mathbf{U}$, stretches/compresses by a factor of $\sigma _{\alpha }$ along the corresponding direction $\hat{\boldsymbol{r}}_{\alpha }$, followed by a rotation aligning with $\hat{\boldsymbol{l}}_{\alpha }$; the left-polar decomposition, $\mathbf{F} = \mathbf{V} \cdot \mathbf{R}$, rotates ${\hat{\boldsymbol{r}}}_{\alpha }$ to align with ${\hat{\boldsymbol{l}}}_{\alpha }$, followed by a stretch/compression by a factor of $\sigma _{\alpha }$ along the corresponding direction ${\hat{\boldsymbol{l}}}_{\alpha }$.

Geometrically, for each $\lambda $, the action of the matrices $\mathbf{V} ( \lambda , \boldsymbol{r}_{0} )$ and $\mathbf{U} ( \lambda , \boldsymbol{r}_{0} )$ is to scale the propagated volume by the singular values of the Spatial Propagator $\mathbf{F} ( \lambda , \boldsymbol{r}_{0} )$, and the action of the rotation matrix $\mathbf{R} ( \lambda , \boldsymbol{r}_{0} )$ is to rotate the singular directions of the reference state $\Omega _{0}$ to align with the singular directions of the propagated state $\Omega _{\lambda }$ (see Figure 5). Thus, *the symmetric matrices* $\mathbf{V} ( \lambda , \boldsymbol{r}_{0} )$ *and* $\mathbf{U} ( \lambda , \boldsymbol{r}_{0} )$*, and orthogonal matrix* $\mathbf{R} ( \lambda , \boldsymbol{r}_{0} )$*, respectively, characterize the smooth anisotropic stretch and rigid-body rotation of the propagated volume along the congruence.*

The basis ${\hat{\boldsymbol{l}}}_{\alpha } ( \lambda , \boldsymbol{r}_{0} )$ describes the singular axes of the stretched and rotated state $\Omega _{\lambda }$. Similarly, for any $\lambda $ the basis ${\hat{\boldsymbol{r}}}_{\alpha } ( \lambda , \boldsymbol{r} _{0} )$ identify the singular axes of the stretched but un-rotated reference state $\Omega _{0}$. Hence, for any $\lambda $ the basis ${\hat{\boldsymbol{r}}}_{\alpha } ( \lambda , \boldsymbol{r} _{0} )$ are the singular directions of the reference state $\Omega _{0}$. We note, however, a spherical reference state $\Omega _{0}$ has no preferred directions; this follows from the fact that the initial condition $\mathbf{F} ( 0, \boldsymbol{r}_{0} ) = \mathbf{I}$ possesses a single degenerate singular value (eigenvalue) $\sigma = 1$ of multiplicity 3. The resolution of this is that a unique, non-degenerate, orthogonal basis ${\hat{\boldsymbol{r}}}_{\alpha } ( \lambda , \boldsymbol{r}_{0} )$ in the reference state $\Omega _{0}$ only exists and *follows from* a Spatial Propagator $\mathbf{F} ( \lambda , \boldsymbol{r}_{0} )$ with non-degenerate singular values.

From a computational standpoint, since $\mathbf{F}$ is non-singular, the matrices $\mathbf{V}$, $\mathbf{U}$, and $\mathbf{R}$ may be determined directly from $\mathbf{F}$, $\mathbf{F} \cdot \mathbf{F}^{T}$, and $\mathbf{F}^{T} \cdot \mathbf{F}$. Using Equations 38 and 39, and that $\mathbf{R}$ is an orthogonal matrix, $\mathbf{V}$, $\mathbf{U}$, and $\mathbf{R}$ follow immediately:

46 $$\begin{aligned} \mathbf{V} ( \lambda , \boldsymbol{r}_{0} ) =& \sqrt{\ \mathbf{F} ( \lambda , \boldsymbol{r}_{0} ) \cdot \mathbf{F}^{T} ( \lambda , \boldsymbol{r}_{0} ) }, \\ \mathbf{U} ( \lambda , \boldsymbol{r}_{0} ) =& \sqrt{ \mathbf{F}^{T} ( \lambda , \boldsymbol{r}_{0} ) \cdot \mathbf{F} ( \lambda , \boldsymbol{r}_{0} ) }, \\ \mathbf{R} ( \lambda , \boldsymbol{r}_{0} ) =& \mathbf{F} ( \lambda , \boldsymbol{r}_{0} ) \cdot \mathbf{U}^{-1} ( \lambda , \boldsymbol{r}_{0} ) = \mathbf{V}^{-1} ( \lambda , \boldsymbol{r}_{0} ) \cdot \mathbf{F} ( \lambda , \boldsymbol{r}_{0} ). \end{aligned}$$

Since the matrices $\mathbf{F} \cdot \mathbf{F}^{T}$ and $\mathbf{F} ^{T} \cdot \mathbf{F}$ are diagonalizable with respect to their respective eigen-bases, the square-root operation is well defined.

It may be shown the full $3 \times 3$ matrix representation of the Spatial Propagator $\mathbf{F} ( \lambda , \boldsymbol{r}_{0} )$ is *directly dependent* on the current distribution. Consider a quasi-static magnetic field $\boldsymbol{B} ( \boldsymbol{r} )$ with a non-trivial current distribution $\boldsymbol{J} ( \boldsymbol{r} )$ that satisfies Ampere’s Law: $\mu _{0} \boldsymbol{J} ( \boldsymbol{r} ) = \nabla \times \boldsymbol{B} ( \boldsymbol{r} )$. The covariant differential of the vector field $\nabla \boldsymbol{B}$ may be decomposed into symmetric and anti-symmetric parts,

47 $$\begin{aligned} ( \nabla \boldsymbol{B} \vert _{\boldsymbol{r}} ){}^{i} {}_{j} =& \frac{1}{2} \bigl( ( \nabla \boldsymbol{B} \vert _{\boldsymbol{r}} ){}^{i}{}_{j} + \bigl( \nabla \boldsymbol{B} \vert _{\boldsymbol{r}}{}^{T} \bigr){}^{i}{}_{j} \ \bigr) + \frac{\mu _{0}}{2} \bigl( \mathbf{J}^{\times } \vert _{\boldsymbol{r}} \bigr){}^{i}{}_{j}. \end{aligned}$$

where the superscript $T$ denotes the matrix transpose. The matrix $\mathbf{J}^{\times } ( \boldsymbol{r} )$, denoted “J-supercross”, is an antisymmetric, $3 \times 3$ matrix representation of the current distribution (with respect to a Cartesian basis),

48 $$\begin{aligned} \bigl( \mathbf{J}^{\times } \vert _{\boldsymbol{r}} \bigr){}^{i} {}_{j} \equiv & \left ( \textstyle\begin{array} {c@{\quad }c@{\quad }c} 0 & J_{z} ( \boldsymbol{r} ) & -J_{y} ( \boldsymbol{r} ) \\ -J_{z} ( \boldsymbol{r} ) & 0 & J_{x} ( \boldsymbol{r} ) \\ J_{y} ( \boldsymbol{r} ) & -J_{x} ( \boldsymbol{r} ) & 0 \end{array}\displaystyle \ \right ). \end{aligned}$$

The Spatial Propagator governing matrix ODE Equation 16 may be written

49 $$\begin{aligned} \frac{\partial ( \mathbf{F} \vert _{\lambda , \boldsymbol{r}_{0}} \ ){}^{i}{}_{j}}{\partial \lambda } =& \frac{1}{2} \sum _{k} \bigl( ( \nabla \boldsymbol{B} \vert _{\boldsymbol{r}} ) {}^{i}{}_{k} + \bigl( \nabla \boldsymbol{B} \vert _{\boldsymbol{r}} {}^{T} \bigr){}^{i}{}_{k} + \mu _{0} \bigl( \mathbf{J}^{\times } \vert _{\boldsymbol{r}} \bigr){}^{i}{}_{k} \bigr) ( \mathbf{F} \vert _{\lambda , \boldsymbol{r}_{0}} ){}^{k}{}_{j}. \end{aligned}$$

The initial condition Equation 17 remains unchanged. We note that dropping the quasi-steady assumption leads to a similar ODE with the addition of a “supercross” displacement-current-density matrix term; we do not pursue such high-frequency dynamics here.

Since the left-stretch $\mathbf{V} ( \lambda , \boldsymbol{r}_{0} )$ and right-stretch $\mathbf{U} ( \lambda , \boldsymbol{r}_{0} )$ matrices are symmetric, through Equation 49, the rotation matrix $\mathbf{R} ( \lambda , \boldsymbol{r}_{0} )$ implicitly requires a non-trivial anti-symmetric part of the covariant differential $\nabla \boldsymbol{B} ( \boldsymbol{r} )$, equivalently a non-trivial (parallel) current distribution $\boldsymbol{J} ( \boldsymbol{r} )$. Moreover, under reasonable conditions we identify a class of magnetic fields $\boldsymbol{B} ( \boldsymbol{r} )$ for which the rotation matrix $\mathbf{R} ( \lambda , \boldsymbol{r}_{0} )$ depends *only* on the parallel current distribution $\boldsymbol{J} ( \boldsymbol{r} )$. Geometrically, the congruence rotation describes the twist of all neighboring integral curves about a central axis field line, and is therefore a measure of the twist helicity. The relationship between the Spatial Propagator, rotation matrix, (twist-) magnetic helicity, and (parallel) current distribution is beyond the scope of this work.

### Quasi-Separatrix Layers and the $Q$-Factor from the Spatial Propagator

We have shown in the previous Sections 3.1 and 3.2 that the congruence geometry is equivalent to the kinematics of a volume undergoing dilation, stretch deformation, and rigid-body rotation under the action of the Spatial Propagator $\mathbf{F} ( \lambda , \boldsymbol{r}_{0} )$. Hence, a separatrix surface reflects the total-collapse of the 3D volume into a 2D surface as it is propagated by the congruence. The quasi-separatrix layer is similarly identified by an “extreme” kinematic deformation of the 3D volume; “extreme” being a subjective term and requiring a (user-defined) quantified threshold.

We remark, recalling from Section 3.1 that the divergence-free condition for vector magnetic fields leads to the topological invariant $\operatorname{det} \mathbf{F} ( \lambda , \boldsymbol{r}_{0} ) = 1$. Geometrically, the volumetric kinematics are incompressible;^5^ that is, the 3D shape deforms under the action of $\mathbf{F} ( \lambda , \boldsymbol{r}_{0} )$ while the total volume remains constant. Hence, the separatrix surfaces in a vector magnetic field are described by infinite stretch deformation of the volume.

For a fixed reference point $\boldsymbol{r}_{0}$, the 3D stretch deformation of a congruence at each $\lambda $ is quantified by the three non-negative singular values $\sigma _{\alpha } \in \mathbb{R} _{\geq 0}$ of the Spatial Propagator $\mathbf{F} ( \lambda , \boldsymbol{r}_{0} )$. There is an inter-dependence between the singular values that is governed by the divergence-free condition. The relative magnitudes of the singular values effectively reflect the $\nabla \mathbf{B}$ matrix eigenvalue structure; the full algebraic proof is beyond the scope of this work. Hence, from a geometric perspective, the relative strengths of the set of singular values may be used to identify 2D separatrix surfaces, 1D separator lines, and 0D null points within the field structure. These topological features are identified by the basic geometric interpretations of the singular values following from the following features.

i) If any one singular value becomes zero, $\sigma _{i} \to 0$, then the other two correspondingly diverge to infinity, $\sigma _{j}, \sigma _{k} \to \infty $; geometrically, the 3D field-line bundle has collapsed into a 2D surface.
ii) If any two of the singular values become zero, $\sigma _{i}, \sigma _{j} \to 0$, then the remaining one correspondingly diverges to infinity, $\sigma _{k} \to \infty $; geometrically, the 3D field-line bundle has collapsed into a 1D line.
iii) The divergence-free condition prevents all three singular values from approaching zero simultaneously; geometrically, the 3D incompressible field-line bundle cannot collapse to a 0D point.

For example, in the natural representation the fan surface and spine line(s) associated with a magnetic null are identified in the limit as $\vert \lambda \vert \to \infty $ by the vanishing of one and two singular values, respectively; the sign of $\lambda $ depends on the field-line polarity. More generally, however, the identification of other non-trivial topological features that exhibit the vanishing of one, or multiple, singular values at finite and/or infinite $\vert \lambda \vert $ remains an open question.

Truly infinite singular values $\sigma _{\alpha } \to \infty $ determine 2D separatrix surfaces and 1D separator lines. “Extreme” singular values identify quasi-separatrix layers (QSLs); that is, *e.g.*, $\sigma _{i} \leq 1/\epsilon $ and correspondingly $\sigma _{j}, \sigma _{k} \geq \epsilon $, for some user-defined threshold $\epsilon \gg 1$. Analogous “extreme” threshold arguments may be made around separator lines.

Up to this point, we have investigated the geometric meaning, consequences, and relations between the singular values of the full-3D Spatial Propagator in various limiting cases. The QSL is often described in terms of the “squashing” of a flux tube (see, *e.g.*, Titov, 2007; Tassev and Sevcheva, 2017; Scott, Pontin, and Hornig, 2017); essentially, a relational measure between the eccentricity of the flux-tube cross-sectional area at the system boundaries, and hence is inherently 2D. Moreover, the squashing of a flux tube is defined entirely independent of rotation. For the remainder of this section, we demonstrate the dimensional reduction of the full-3D Spatial Propagator to describe the 2D squashing of the congruence. In particular, we illustrate a simple construction of the popular squashing factor $Q$ (Titov, Hornig, and Démoulin, 2002; Titov, 2007), from the Spatial Propagator. We note, our aim here is *neither* a reformulation, nor a more efficient computation of the squashing factor $Q$ over that available in the open literature (see, *e.g.*, Tassev and Sevcheva, 2017). Rather the purpose is simply to show exactly how the popular $Q$-value may be derived from, and therefore fits within, the general Spatial Propagator framework.

Throughout this article we have assumed that the vector magnetic field $\boldsymbol{B} ( \boldsymbol{r} )$ has smooth component functions, Equation 1, that may be described with respect to a single global Cartesian coordinate chart. Hence the congruence solution components, Equation 19, described with respect to the same global Cartesian coordinate chart, are also smooth, single-valued functions of the connectivity parameter $\lambda $, and reference condition coordinates $\boldsymbol{r}_{0}$. Furthermore, by construction, this framework is valid for any global coordinate chart that covers the system (see Appendix B.1 for general coordinate chart formulations, and Appendices B.2 and B.3 for spherical–polar formulations).

Recall from Section 2.1 that we identify $\lambda = 0$ with the reference point $\boldsymbol{r}_{0}$, corresponding to the beginning of the congruence, and we identify the *fixed, finite* value $\lambda = L$ with $\boldsymbol{r} = \boldsymbol{r} ( L, \boldsymbol{r}_{0} )$, corresponding to the end of the congruence. In general, we take the reference point $\boldsymbol{r}_{0}$ and end point $\boldsymbol{r} ( L, \boldsymbol{r}_{0} )$ on the system boundary.^6^ From this perspective, *each particular field-line solution with* $L > 0$ *provides a unique connectivity map between disjoint points on the system boundary, and the Spatial Propagator quantifies the local geometric organization of this connectivity map within its characteristic scale*.

**Figure 6:**
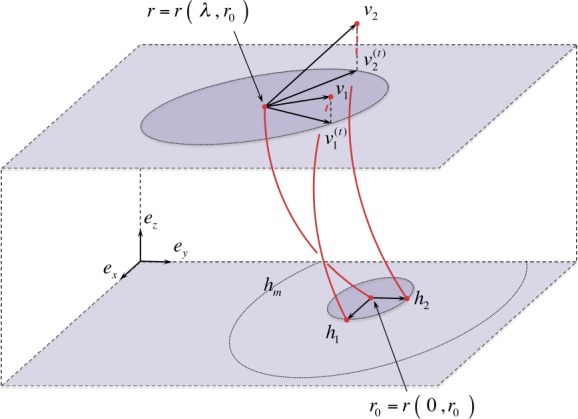
Illustration of the QSL geometry. Simple example with top (bottom) boundaries at $z = \pm L_{z}$. An initial circle defined by $\lbrace \boldsymbol{h}_{1}, \boldsymbol{h}_{2} \rbrace $ deforms into an ellipsoid defined by the projections $\lbrace \boldsymbol{v}^{(t)}_{1}, \boldsymbol{v}^{(t)}_{2} \rbrace $ of the propagated vectors $\lbrace \boldsymbol{v}_{1}, \boldsymbol{v}_{2} \rbrace $ onto the plane $z = L_{z}$.

Consider for simplicity a smooth vector field $\boldsymbol{B} ( \boldsymbol{r} )$ in a bounded Cartesian box; that is, we let the coordinate domains be $x \in [ -L_{x}, L_{x} ]$, $y \in [ -L_{y}, L_{y} ]$, and $z \in [ -L_{z}, L _{z} ]$. The (inward) unit normal $\hat{\boldsymbol{n}}$ at the boundaries is parallel (up to a sign) to the respective Cartesian basis vectors $\lbrace \hat{\boldsymbol{e}}_{x}, \hat{\boldsymbol{e}}_{y}, \hat{\boldsymbol{e}}_{z} \rbrace $. Moreover, consider a congruence solution, $\boldsymbol{r} ( \lambda , \boldsymbol{r}_{0} )$ and $\mathbf{F} ( \lambda , \boldsymbol{r}_{0} )$ (Equation 19) with characteristic scale $h_{m}$, consistent with the given vector field $\boldsymbol{B} ( \boldsymbol{r} )$ such that the reference point $\boldsymbol{r}_{0}$ is chosen at the $z = -L_{z}$ boundary, and the end point $\boldsymbol{r} ( L, \boldsymbol{r}_{0} )$ follows on the $z = L_{z}$ boundary (see Figure 6).

At $\boldsymbol{r}_{0}$, the boundary surface $z = -L_{z}$ has a unit normal $\hat{\boldsymbol{n}} ( \boldsymbol{r}_{0} ) = \hat{\boldsymbol{e}}_{z}$. We may construct two orthogonal reference shift vectors $\lbrace \boldsymbol{h}_{1}, \boldsymbol{h}_{2} \rbrace $ tangent to the $z = -L_{z}$ boundary plane; that is, both $\lbrace \boldsymbol{h}_{1}, \boldsymbol{h}_{2} \rbrace $ satisfy a tangency constraint, $\boldsymbol{h}_{\alpha } \cdot \hat{\boldsymbol{n}} ( \boldsymbol{r}_{0} ) = 0$ for each $\alpha = 1, 2$. Without loss of generality, we may choose the $\lbrace \boldsymbol{h}_{1}, \boldsymbol{h}_{2} \rbrace $ proportional to the basis vectors $\lbrace \hat{\boldsymbol{e}}_{x}, \hat{\boldsymbol{e}}_{y} \rbrace $. Written with respect to the global $\lbrace \hat{\boldsymbol{e}}_{x}, \hat{\boldsymbol{e}}_{y}, \hat{\boldsymbol{e}}_{z} \rbrace $ basis, the reference shift vectors are

50 $$\begin{aligned} \begin{aligned} \boldsymbol{h}_{1} &=& \bigl( \vert \boldsymbol{h}_{1} \vert \bigr) {}^{x} \hat{\boldsymbol{e}}_{x}, \\ \boldsymbol{h}_{2} &=& \bigl( \vert \boldsymbol{h}_{2} \vert \bigr) {}^{y} \hat{\boldsymbol{e}}_{y}. \end{aligned} \end{aligned}$$

Moreover, the components of each reference shift vector $\boldsymbol{h} _{\alpha }$ are subject to the characteristic scale constraint; for $\alpha = 1, 2$,

51 $$\begin{aligned} \vert \boldsymbol{h}_{\alpha } \vert =& \sqrt{ \sum _{i} ( \boldsymbol{h}_{\alpha } ){}^{i} ( \boldsymbol{h}_{ \alpha } ){}^{i} } \ll h_{m}. \end{aligned}$$

where $h_{m}$ is defined in Appendix A.

By Equation 12, these reference shift vectors $\lbrace \boldsymbol{h}_{1}, \boldsymbol{h}_{2} \rbrace $ are propagated along the congruence to the target footpoint $\boldsymbol{r} ( L, \boldsymbol{r}_{0} )$. The propagated vectors at $\lambda = L$ are also written with respect to the global $\lbrace \hat{\boldsymbol{e}} _{x}, \hat{\boldsymbol{e}}_{y}, \hat{\boldsymbol{e}}_{z} \rbrace $ basis,

52 $$\begin{aligned} \begin{aligned} \boldsymbol{v}_{1} ( L , \boldsymbol{r}_{0} ) &=& \sum_{i} ( \mathbf{F} \vert _{L , \boldsymbol{r}_{0}} ){}^{i} {}_{x} \bigl( \vert \boldsymbol{h}_{1} \vert \bigr){}^{x} \hat{\boldsymbol{e}}_{i}, \\ \boldsymbol{v}_{2} ( L , \boldsymbol{r}_{0} ) &=& \sum _{i} ( \mathbf{F} \vert _{L , \boldsymbol{r}_{0}} ){}^{i} {}_{y} \bigl( \vert \boldsymbol{h}_{2} \vert \bigr){}^{y} \hat{\boldsymbol{e}}_{i}. \end{aligned} \end{aligned}$$

Since by assumption the vector field $\boldsymbol{B} ( \boldsymbol{r} )$ is everywhere smooth, the Spatial Propagator $\mathbf{F} ( \lambda , \boldsymbol{r}_{0} )$ is non-singular for all $\lambda $, and hence all linearly independent $\lbrace \boldsymbol{h}_{1}, \boldsymbol{h}_{2} \rbrace $ are mapped to linearly independent $\lbrace \boldsymbol{v}_{1} ( L , \boldsymbol{r}_{0} ), \boldsymbol{v}_{2} ( L , \boldsymbol{r}_{0} ) \rbrace $. Furthermore, while we have the freedom to choose the reference shift vectors $\boldsymbol{h}_{\alpha }$ tangent to the boundary surface at the launch footpoint $\boldsymbol{r}_{0}$, in general the propagated vectors $\boldsymbol{v}_{\alpha } ( L , \boldsymbol{r}_{0} )$ at the target footpoint $\boldsymbol{r} ( L, \boldsymbol{r}_{0} )$ are *not* tangent to their respective boundary surface. However, we may project the $\boldsymbol{v}_{\alpha } ( L , \boldsymbol{r}_{0} )$ onto the tangent boundary plane (see Figure 6)

53 $$\begin{aligned} \boldsymbol{v}^{(t)}_{\alpha } ( L , \boldsymbol{r}_{0} ) =& \bigl( \mathbf{I} - \hat{\boldsymbol{n}} ( \boldsymbol{r} ) \otimes \hat{\boldsymbol{n}} ( \boldsymbol{r} ) \bigr) \cdot \boldsymbol{v}_{\alpha } ( L , \boldsymbol{r}_{0} ), \end{aligned}$$

with $r = \boldsymbol{r} ( L, \boldsymbol{r}_{0} )$ at the target footpoint. The superscript $(t)$ denotes the projection tangent to the boundary. In our simple example, at the target footpoint, $\boldsymbol{r} = \boldsymbol{r} ( L, \boldsymbol{r}_{0} )$, the relation $\hat{\boldsymbol{n}} ( \boldsymbol{r} ) = - \hat{\boldsymbol{e}}_{z}$ and Equation 53 yield

54 $$\begin{aligned} \begin{aligned} \boldsymbol{v}^{(t)}_{1} ( L , \boldsymbol{r}_{0} ) &= ( \mathbf{F} \vert _{L , \boldsymbol{r}_{0}} ){}^{x} {}_{x} \bigl( \vert \boldsymbol{h}_{1} \vert \bigr){}^{x} \hat{ \boldsymbol{e}}_{x} + ( \mathbf{F} \vert _{L , \boldsymbol{r} _{0}} ){}^{y}{}_{x} \bigl( \vert \boldsymbol{h}_{1} \vert \bigr){}^{x} \hat{\boldsymbol{e}}_{y} , \\ \boldsymbol{v}^{(t)}_{2} ( L , \boldsymbol{r}_{0} ) &= ( \mathbf{F} \vert _{L , \boldsymbol{r}_{0}} ){}^{x} {}_{y} \bigl( \vert \boldsymbol{h}_{2} \vert \bigr){}^{y} \hat{ \boldsymbol{e}}_{x} + ( \mathbf{F} \vert _{L , \boldsymbol{r} _{0}} ){}^{y}{}_{y} \bigl( \vert \boldsymbol{h}_{2} \vert \bigr){}^{y} \hat{\boldsymbol{e}}_{y}. \end{aligned} \end{aligned}$$

We remark that the construction/projection procedure Equation 53 is valid for any surface with normal $\hat{\boldsymbol{n}} ( \boldsymbol{r} )$ on the system boundary.

The local properties of the mapping of vectors $\boldsymbol{h}_{\alpha }$ tangent to the boundary plane at $\boldsymbol{r}_{0}$, into vectors $\boldsymbol{v}^{(t)}_{\alpha } ( L , \boldsymbol{r}_{0} )$ tangent to the boundary plane at $\boldsymbol{r} = \boldsymbol{r} ( L, \boldsymbol{r}_{0} )$ are described by the $2 \times 2$ matrix $\mathbf{D}$; the components of which are

55 $$\begin{aligned} ( \mathbf{D} ){}^{i}{}_{j} \equiv & \frac{ ( \boldsymbol{v}^{(t)}_{\alpha } \vert _{L , \boldsymbol{r}_{0}} ) {}^{i}}{ ( \boldsymbol{h}_{\alpha } ){}^{j}} = \left ( \textstyle\begin{array} {c@{\quad }c} ( \mathbf{F} \vert _{L , \boldsymbol{r}_{0}} ){}^{x} {}_{x} & ( \mathbf{F} \vert _{L , \boldsymbol{r}_{0}} ) {}^{y}{}_{x} \\ ( \mathbf{F} \vert _{L , \boldsymbol{r}_{0}} ){}^{x} {}_{y} & ( \mathbf{F} \vert _{L , \boldsymbol{r}_{0}} ) {}^{y}{}_{y} \end{array}\displaystyle \right ). \end{aligned}$$

The matrix $\mathbf{D}$ is the sub-matrix of $\mathbf{F} ( L, \boldsymbol{r}_{0} )$ obtained by removing the column corresponding to the coordinate direction parallel to the launch-surface normal and row corresponding to the coordinate direction parallel to the target-surface normal; in this example, remove the column $( \mathbf{F} \vert _{L , \boldsymbol{r}_{0}} ){}^{z}{}_{j}$ and remove the row $( \mathbf{F} \vert _{L , \boldsymbol{r}_{0}} ) {}^{i}{}_{z}$. This principle is the same for general curvilinear coordinate systems, where general boundary-surface unit normals are not parallel to coordinate basis directions at all positions on the boundary surface. Hence the tensorial nature of the Spatial Propagator allows one simply to rotate the proper components of the $3 \times 3$ matrix into coordinates parallel to the surface normals at the point on appropriate boundaries, and then remove the appropriate rows/columns to construct the proper $2 \times 2$ sub-matrix; we leave such analysis for future work.

The field lines define a map from $h$-coordinates with origin at $\boldsymbol{r}_{0}$, to $v^{(t)}$-coordinates with origin at $\boldsymbol{r} = \boldsymbol{r} ( L, \boldsymbol{r}_{0} )$. By Equations 16 and 17, the basis vectors $\boldsymbol{h}_{\alpha }$ are propagated, via Lie transport (Scott, Pontin, and Hornig, 2017), from $\boldsymbol{r}_{0}$ to $\boldsymbol{r} = \boldsymbol{r} ( L, \boldsymbol{r}_{0} )$. The matrix $\mathbf{D}$ may be interpreted as the Jacobian of this map, given by the corresponding components of the matrix representation of the Spatial Propagator $( \mathbf{F} \vert _{L , \boldsymbol{r}_{0}} ) {}^{i}{}_{j}$. The squashing factor $Q$, may be immediately constructed; in the notation of Titov, Hornig, and Démoulin (2002),

56 $$ Q = \frac{N^{2}}{\Delta }, $$

where the norm $N$ (Démoulin *et al.*, 1996) is given by

57 $$\begin{aligned} N^{2} =& \bigl( ( \mathbf{F} \vert _{L , \boldsymbol{r}_{0}} ){}^{x}{}_{x} \bigr){}^{2} + \bigl( ( \mathbf{F} \vert _{L , \boldsymbol{r}_{0}} ){}^{y}{}_{y} \bigr){}^{2} + \bigl( ( \mathbf{F} \vert _{L , \boldsymbol{r}_{0}} ) {}^{x}{}_{y} \bigr){}^{2} + \bigl( ( \mathbf{F} \vert _{L , \boldsymbol{r}_{0}} ){}^{y}{}_{x} \bigr){}^{2}, \end{aligned}$$

and $\Delta $ is the determinant of the Jacobian matrix $\mathbf{D}$,

58 $$\begin{aligned} \Delta =& ( \mathbf{F} \vert _{L , \boldsymbol{r}_{0}} ) {}^{x}{}_{x} ( \mathbf{F} \vert _{L , \boldsymbol{r}_{0}} ) {}^{y}{}_{y} - ( \mathbf{F} \vert _{L , \boldsymbol{r}_{0}} ) {}^{x}{}_{y} ( \mathbf{F} \vert _{L , \boldsymbol{r}_{0}} ) {}^{y}{}_{x}. \end{aligned}$$

In general, Equation 58 is the minor of the sub-matrix corresponding to the $( \mathbf{F} \vert _{L , \boldsymbol{r} _{0}} ){}^{i}{}_{j}$ matrix component, where the $i$-index corresponds to the coordinate direction parallel to the launch-surface normal, and the $j$-index corresponds to the coordinate direction parallel to the target-surface normal.

Moreover, the eigenvalues $\lbrace d_{1}, d_{2} \rbrace $ of the $2 \times 2$ Jacobian matrix $\mathbf{D}$, given by the roots of the characteristic equation

$$\begin{aligned} \operatorname{det} ( \mathbf{D} - d \mathbf{I} ) =& 0, \end{aligned}$$

are related to the $Q$-value via

59 $$\begin{aligned} \frac{\vert d_{1} \vert }{\vert d_{2} \vert } =& \frac{Q}{2} + \sqrt{ \biggl( \frac{Q}{2} \biggr)^{2} -1 }, \end{aligned}$$

which, after some algebra, reduces to

60 $$\begin{aligned} Q = \frac{d_{1}}{d_{2}} + \frac{d_{2}}{d_{1}}. \end{aligned}$$

We remark that *the line-tying condition does not imply a fixed Spatial Propagator; only the proper sub-matrices and their combinatorics forming the squashing factor* $Q$ *are preserved.*

The $Q$-Map (Titov, 2007), identifies the separatrix and QSL field structures. However, on their own, these regions offer only the *possible* current-sheet formation sites in the field structure. Whether or not electromagnetic stresses accumulate resulting in subsequent current-sheet formation depends on the details of energization and stress injection; *e.g.* separatrix and QSL structures undergoing rigid-body motion will not develop a current sheet (Aulanier, Pariat, and Démoulin, 2005; Aulanier *et al.*, 2010; Janvier *et al.*, 2014); whereas more general motions, such as shearing or twisting motions, will develop a current sheet unstable to reconnection (Aulanier *et al.*, 2006; Effenberger *et al.*, 2011; Janvier *et al.*, 2013).

## Conclusion and Future Applications

In this article we introduce a generalized field-line connectivity phase space associated with the vector magnetic field in which the geometric and topological features of the system are made explicit. The fundamental assumption is that the vector magnetic field is *a priori* smooth everywhere. The basic elements are the field line and its linearized variation, the Spatial Propagator: Equation 19. Equations 2 and 16, with initial conditions from Equations 3 and 17, provide a direct formulation of these phase-space elements in terms of the vector magnetic field and its spatial derivatives. Furthermore, the field line and Spatial Propagator are constructed with respect to general curvilinear coordinates and the equivalence class of general affine parameterizations.

The geometric interpretation is that the Spatial Propagator characterizes the organization of the local bundle of field lines. Since the vector field is everywhere smooth, so too are the field-line and Spatial Propagator solutions smooth and unique. The geometric organization of the local bundle is completely equivalent to a kinematic description of a volume centered on the particular field-line solution and undergoing deformation by transport along the field. This deformation kinematics is characterized by volumetric dilation, anisotropic stretch, and rotation. The volumetric dilation (expansion/compression) is completely described by the determinant of the Spatial Propagator: Equation 30. For the vector magnetic field, the determinant of the Spatial Propagator is a topological invariant everywhere equal to unity, Equation 34, which reflects the divergence-free condition. The anisotropic stretch and rotation kinematics are described by the singular values and singular vectors of the Spatial Propagator. The singular values, Equation 37, characterize the general anisotropic stretch of the congruence volume. The congruence rotation is a simple rigid-body rotation kinematics between the orthonormal principal-direction bases: Equation 43, from the reference volume $\Omega _{0}$ to the propagated volume $\Omega _{\lambda }$.

Extreme singular values identify QSLs within the system; true separatrix surfaces and separator lines within the system are identified in the limiting cases of one, or two, zero singular values, respectively. Moreover, the $Q$-factor is simply constructed from analysis of the particular sub-matrix of the Spatial Propagator obtained by removing the column corresponding to the coordinate direction parallel to the launch-surface normal and row corresponding to the coordinate direction parallel to the target-surface normal.

This magnetic connectivity phase-space framework opens up extensive directions in geometric and topological analysis of vector magnetic fields. For example, in future work we will relax the *a priori* smooth vector magnetic-field assumption in order to analyze both existing singular structures such as current sheets and their formation. Moreover, the magnetic helicity may be decomposed into twist and writhe components (Moffatt and Ricca, 1992); in future efforts we will show that, accounting for relative shearing of the volume, twist helicity may be described with the congruence rotation, while the writhe helicity is related to the embedding of the field-line trajectory and propagation of the reference shift vector in 3D space.

In the present article the field-line bundle and Spatial Propagator are presented and analyzed from a geometric perspective. Since the field lines are curves (*i.e.* *spatial positions*) in a three-dimensional space whose tangent vector is everywhere parallel to the magnetic-field vector, evolutionary dynamics (ideal or otherwise) of the field-line bundle are often described by imposing the frozen-in condition (*e.g.* infinite plasma conductivity) somewhere within the system domain (typically at the system boundary). This allows one to describe and follow the dynamics, not of the field line itself, but rather of the parcel of plasma to which the field line is connected. In non-ideal systems; the difference between the ideal motion and the actual motion of the field is attributed to resistive slipping; this is the origin of “slip-running reconnection” and other resistive slip phenomenology. In follow-up analysis, we use this formalism to describe the dynamics of a field-line bundle and associated Spatial Propagator without reference to plasma conductivity, or material parcels, but rather with respect to pure electric and magnetic fields; such a description necessarily requires a treatment of the full four-dimensional electromagnetic-field tensor.

In particular, we will show that the magnetic field generalizes to the antisymmetric, dual electromagnetic-field tensor $\ast \mathcal{F} ^{\mu \nu } ( t, \boldsymbol{x} )$ (see, *e.g.*, Jackson, 1999, Section 11.9). The field line generalizes to a 4-vector flow field $\phi ^{\mu } ( \lambda ; t_{0}, \boldsymbol{r}_{0} )$, where the spatial components are identified with the canonical notion of a field line. In addition, the propagator generalizes to a second-order mixed tensor field $F^{ \mu }{}_{\nu } ( t, \boldsymbol{x} )$ where the spatial components are identified with the Spatial Propagator described in this article. Moreover, we will show that expanding these four-dimensional generalizations into explicit spatial and temporal components recovers the definition of a magnetic-field line as three *spatial constraint* equations,

61 $$\begin{aligned} ( \boldsymbol{B} \cdot \nabla ) \phi ^{i} =& B^{i}, \end{aligned}$$

and three *time-dynamic* equations for the field-line components,

62 $$\begin{aligned} \frac{B^{2}}{4 \pi } \frac{\partial \phi ^{i}}{\partial t} + ( \boldsymbol{S} \cdot \nabla ) \phi ^{i} =& S^{i}. \end{aligned}$$

where $\boldsymbol{S}$ is the Poynting vector (Jackson, 1999, pp. 608 – 610). An explicit spatial and temporal decomposition of the propagator follows similarly.

Finally, this framework allows for the analysis of extrinsic thermodynamic properties, such as the total mass or total magnetic energy, of the field-line bundle through Equations 31 and 32. In particular, we derive an analogous first law of thermodynamics applied to the pure magnetic-congruence geometry,

63 $$\begin{aligned} \Delta E ( L, 0; \Omega _{0} ) =& E ( L, \Omega _{0} ) - E ( 0, \Omega _{0} ) \\ =& \int _{0}^{L} \int _{\Omega _{0}} \biggl( \operatorname{Tr} \bigl( \mathbf{T} \cdot ( \nabla \boldsymbol{B} ) \bigr) + \rho \boldsymbol{E} \cdot \boldsymbol{B} + \frac{1}{c^{2}} \boldsymbol{E} \cdot ( \nabla \times \boldsymbol{S} ) \biggr) \,\mathrm{d}^{3} \boldsymbol{x} \,\mathrm{d}\lambda , \end{aligned}$$

where $\boldsymbol{E}$ and $\boldsymbol{B}$ are the electric and magnetic fields, $\mathbf{T}$ is the matrix representation of the electromagnetic stress tensor, $\rho $ is the net charge density, and $\boldsymbol{S}$ is the Poynting vector (Jackson, 1999, pp. 608 – 610); all quantities are evaluated along the field line $\boldsymbol{r} = \boldsymbol{r} ( \lambda , \boldsymbol{r}_{0} )$. Equation 63 is the total electromagnetic energy of a congruence formulated in terms analogous to mechanical work and energy generation. We will derive Equation 63 and explore applications to coronal phenomenology (*e.g.* coronal heating, active-region stability, flares, and CME initiation) in future work.

## Appendix A: Characteristic Scale $h_{m}$ of the Spatial Propagator

In this appendix, we derive the local characteristic scale $h_{m}$ over which the Spatial Propagator $\mathbf{F} ( \lambda , \boldsymbol{r}_{0} )$ describes the relative behavior of field lines within a congruence. The family of integral curve solutions $\boldsymbol{r} ( \lambda , \boldsymbol{r}_{0} )$ to the system of Equations 2 and 3 are written in components with respect to a generally valid global basis $\boldsymbol{e}_{i}$ (see Appendix B.1),

64 $$\begin{aligned} \boldsymbol{r} ( \lambda , \boldsymbol{r}_{0} ) =& \sum _{i} ( \boldsymbol{r} \vert _{\lambda , \boldsymbol{r}_{0}} ) {}^{i} \boldsymbol{e}_{i} \end{aligned}$$

where the component functions $( \boldsymbol{r} \vert _{\lambda , \boldsymbol{r}_{0}} ){}^{i}$ are smooth functions of the connectivity parameter $\lambda $ and reference condition coordinates $\boldsymbol{r}_{0}$.

Consider two spatially neighboring field lines within the congruence: $\boldsymbol{r} ( \lambda , \boldsymbol{r}_{0} )$ and $\boldsymbol{r} ( \lambda , \boldsymbol{r}_{0} + \boldsymbol{h} )$. We may relate the neighboring field lines via a Taylor expansion of the component functions,

65 $$\begin{aligned} &( \boldsymbol{r} \vert _{\lambda , \boldsymbol{r}_{0} + \boldsymbol{h}} ){}^{i} - ( \boldsymbol{r} \vert _{\lambda , \boldsymbol{r}_{0}} ){}^{i} = \sum_{j} ( \mathbf{F} \vert _{\lambda , \boldsymbol{r}_{0}} \ ){}^{i}{}_{j} h^{j} + \frac{1}{2} \sum_{j,k} ( \mathbf{M} \vert _{\lambda , \boldsymbol{r}_{0}} ){}^{i}{}_{jk} h^{j} h^{k} + \mathrm{O} \bigl( \vert \boldsymbol{h} \vert ^{3} \bigr). \end{aligned}$$

In the most general case, the congruence solution $\boldsymbol{r} ( \lambda , \boldsymbol{r}_{0} )$ is a non-linear function of the reference point $\boldsymbol{r}_{0}$. In this case, the components (at least one) of the second-order variation are non-trivial; *i.e.* $( \mathbf{M} \vert _{\lambda , \boldsymbol{r}_{0}} ){}^{i}{}_{jk} \ne 0$. Hence, we may define the characteristic scale $h_{m}$ of the Spatial Propagator $\mathbf{F} ( \lambda , \boldsymbol{r}_{0} )$ by considering the relative size of the first- and second-order variational terms.

66 $$\begin{aligned} &\bigl\vert ( \boldsymbol{r} \vert _{\lambda , \boldsymbol{r}_{0} + \boldsymbol{h}} ){}^{i} - ( \boldsymbol{r} \vert _{\lambda , \boldsymbol{r}_{0}} ) {}^{i} \bigr\vert \\ &\quad= \biggl\vert \sum_{j} ( \mathbf{F} \vert _{\lambda , \boldsymbol{r}_{0}} ){}^{i}{}_{j} h^{j} + \frac{1}{2} \sum_{j,k} ( \mathbf{M} \vert _{\lambda , \boldsymbol{r}_{0}} ){}^{i}{}_{jk} h^{j} h ^{k} + \mathrm{O} \bigl( \vert \boldsymbol{h} \vert ^{3} \bigr) \biggr\vert \\ &\quad\leq \biggl\vert \sum_{j} ( \mathbf{F} \vert _{\lambda , \boldsymbol{r}_{0}} ){}^{i}{}_{j} h^{j} \biggr\vert + \biggl\vert \frac{1}{2} \sum_{j,k} ( \mathbf{M} \vert _{\lambda , \boldsymbol{r}_{0}} ){}^{i}{}_{jk} \ h^{j} h^{k} \biggr\vert + \mathrm{O} \bigl( \vert \boldsymbol{h} \vert ^{3} \bigr) \\ &\quad\leq \biggl\vert \sum_{j} ( \mathbf{F} \vert _{\lambda , \boldsymbol{r}_{0}} ){}^{i}{}_{j} \biggr\vert \vert \boldsymbol{h} \vert + \frac{1}{2} \biggl\vert \sum _{j,k} ( \mathbf{M} \vert _{\lambda , \boldsymbol{r}_{0}} ) {}^{i}{}_{jk} \biggr\vert \vert \boldsymbol{h} \vert ^{2} + \mathrm{O} \bigl( \vert \boldsymbol{h} \vert ^{3} \bigr). \end{aligned}$$

Since the vector field $\boldsymbol{B} ( \boldsymbol{r} )$ is assumed smooth everywhere, it may be shown that the $L_{1,2}$ norm of the first- and second-order variation matrices $\Vert \mathbf{F} \Vert $ and $\Vert \mathbf{M} \Vert $ are non-singular; that is, there exists a finite $F, M \in \mathbb{R}$ such that

67 $$\begin{aligned} F \equiv & \Vert \mathbf{F} \vert _{\boldsymbol{r}_{0}} \Vert = \max_{\lambda } \biggl( \sum_{i} \biggl( \sum_{j} ( \mathbf{F} \vert _{\lambda , \boldsymbol{r}_{0}} ){}^{i}{} _{j} \biggr)^{2} \biggr)^{1/2}, \end{aligned}$$

68 $$\begin{aligned} M \equiv & \Vert \mathbf{M} \vert _{\boldsymbol{r}_{0}} \Vert = \max _{\lambda } \biggl( \sum_{i} \biggl( \sum_{j,k} ( \mathbf{M} \vert _{\lambda , \boldsymbol{r}_{0}} ){}^{i}{} _{jk} \biggr)^{2} \biggr)^{1/2}. \end{aligned}$$

Hence, Equation 66 becomes

69 $$\begin{aligned} \bigl\vert ( \boldsymbol{r} \vert _{\lambda , \boldsymbol{r}_{0} + \boldsymbol{h}} ){}^{i} - ( \boldsymbol{r} \vert _{\lambda , \boldsymbol{r}_{0}} ) {}^{i} \bigr\vert \leq & F \vert \boldsymbol{h} \vert + \frac{M}{2} \vert \boldsymbol{h} \vert ^{2} + O \bigl( \vert \boldsymbol{h} \vert ^{3} \bigr) \\ \leq & F \vert \boldsymbol{h} \vert \biggl( 1 + \frac{M}{2 F} \vert \boldsymbol{h} \vert + O \bigl( \vert \boldsymbol{h} \vert ^{2} \bigr) \biggr) \\ \leq & F \vert \boldsymbol{h} \vert \biggl( 1 + \frac{\vert \boldsymbol{h} \vert }{h_{m}} + O \bigl( \vert \boldsymbol{h} \vert ^{2} \bigr) \biggr), \end{aligned}$$

where we defined the characteristic scale $h_{m} \equiv 2 F / M$; that is, the characteristic scale is the minimum scale such that the linear term is at least of the same magnitude as the second-order term. Then, for all $\vert \boldsymbol{h} \vert \ll h_{m}$, the higher-order terms of Equation 69 are all negligible relative to the first-order term, and the first-order variation $\mathbf{F} ( \lambda , \boldsymbol{r}_{0} )$ describes the relative behavior of the congruence.

In the special case that the congruence solution $\boldsymbol{r} ( \lambda , \boldsymbol{r}_{0} )$ is linear in reference point $\boldsymbol{r}_{0}$, then all components of $( \mathbf{M} \vert _{\lambda , \boldsymbol{r}_{0}} ){}^{i}{}_{jk} = 0$ and all higher-order derivatives vanish, and $h_{m} \to \infty $. Hence, for all $\boldsymbol{h} \in \mathbb{R}^{3}$,

70 $$ ( \boldsymbol{r} \vert _{\lambda , \boldsymbol{r}_{0} + \boldsymbol{h}} ){}^{i} - ( \boldsymbol{r} \vert _{\lambda , \boldsymbol{r}_{0}} ){}^{i} = \sum _{j} ( \mathbf{F} \vert _{\lambda , \boldsymbol{r}_{0}} ){}^{i}{} _{j} h^{j}. $$

Furthermore, for completeness, in cases of high symmetry the higher-order variations may vanish through some finite order $n$. In such examples, the characteristic scale is defined as $h_{m} \equiv ( \frac{(n+1)! F}{M^{(n+1)}} )^{1/n}$ with $n \ge 2$, where $M^{(n+1)}$ is the $L_{1,2}$ norm of lowest-order non-zero variation matrix. We do not pursue such cases here.

An extremely popular approach to computational solar and space-plasma physics is the linear interpolation methodologies employed in numerical algorithms for the computation of vector-field components between grid points (see, *e.g.*, Tassev and Sevcheva, 2017). Such approaches simply shift the problem of investigating small-scale dynamics from the physical vector magnetic field on to finer and more complex numerical-grid resolutions. Depending on the size of $h_{m}$ relative to the characteristic scales of the system, this may be a fundamental limitation as an accurate representation of the vector-field structure. The formalism presented here is a fundamentally different approach in which the investigative burden remains on the physical quantities, as opposed to simply increasing the computational expense to handle finer and finer mesh structure.

## Appendix B: Matrix Representation of the Covariant Differential of a Vector Field with Respect to General Basis

In this appendix, we briefly review some aspects of canonical differential geometry (*e.g.* general coordinate charts, basis and dual basis vectors, and the differential operator $\nabla $). In particular, we construct the $3 \times 3$ matrix representation of the covariant differential $\nabla \boldsymbol{B} ( \boldsymbol{r} )$ with respect to general, local curvilinear bases. The term *covariant* is standard nomenclature; in this context meaning the object transforms as a tensor under general coordinate transformations. We construct the matrix representation of the covariant differential $\nabla \boldsymbol{B} ( \boldsymbol{r} )$ with respect to a general coordinate chart (Appendix B.1), followed by respective applications to the spherical–polar curvilinear coordinate basis (Appendix B.2), and the spherical–polar orthonormal basis (Appendix B.3) in common use by the solar and space-physics community (see, *e.g.*, Tassev and Sevcheva, 2017).

We note that this appendix is not intended to be a full exposition of differential geometry. We describe only those aspects necessary and sufficient for the construction of the covariant differential of the vector field in curvilinear coordinates; see Kobayashi and Nomizu (1963) for a full axiomatic approach.

### B.1 General Construction of the Covariant Differential of a Vector Field

#### B.1.1 General Curvilinear Coordinates, Basis and Dual Basis Vectors

Let $M \subseteq \mathbb{R}^{3}$ be a subset of three-dimensional space. Consider a general curvilinear coordinate chart $( M, q )$ such that the position of each point $p \in M$ is described by smooth coordinate functions $q : M \to \mathbb{R}^{3}$,

71 $$\begin{aligned} \boldsymbol{r} ( p ) =& \bigl\lbrace q^{1} ( p ), q^{2} ( p ), q^{3} ( p ) \bigr\rbrace . \end{aligned}$$

For ease of notation, we drop the formal dependence on the point $p \in M$, labeling it with only the coordinates $q^{i}$.

The coordinate chart $( M, q )$ admits a natural set of coordinate basis vectors, $\lbrace \boldsymbol{q}_{1}, \boldsymbol{q}_{2}, \boldsymbol{q}_{3} \rbrace $. The (not necessarily orthonormal) basis vector $\boldsymbol{q}_{i}$ is the unique element of the tangent bundle that acts on the (smooth) position vector by taking its derivative with respect to the coordinate $q^{i}$,

72 $$\begin{aligned} \boldsymbol{q}_{i} \equiv \frac{\partial \boldsymbol{r}}{\partial q^{i}}, \end{aligned}$$

such that the position vector Equation 71 has components written with respect to the $\lbrace \boldsymbol{q}_{1}, \boldsymbol{q}_{2}, \boldsymbol{q}_{3} \rbrace $ basis,

73 $$\begin{aligned} \boldsymbol{r} =& \sum_{j} q^{j} \boldsymbol{q}_{j}. \end{aligned}$$

The coordinate basis vectors $\lbrace \boldsymbol{q}_{1}, \boldsymbol{q} _{2}, \boldsymbol{q}_{3} \rbrace $ are often written explicitly with respect to Cartesian basis set $\lbrace \hat{\boldsymbol{e}}_{x}, \hat{\boldsymbol{e}}_{y}, \hat{\boldsymbol{e}}_{z} \rbrace $. Assume that the general curvilinear coordinates are smoothly related to Cartesian coordinates; that is, there exist smooth coordinate transformation functions,

74 $$\begin{aligned} x^{j} = x^{j} \bigl( q^{i} \bigr), \end{aligned}$$

such that the position vector Equation 71 has components written with respect to the $\lbrace \hat{\boldsymbol{e}} _{x}, \hat{\boldsymbol{e}}_{y}, \hat{\boldsymbol{e}}_{z} \rbrace $ basis

75 $$\begin{aligned} \boldsymbol{r} =& \sum_{j} x^{j} \hat{\boldsymbol{e}}_{j}. \end{aligned}$$

Since the Cartesian basis vectors $\hat{\boldsymbol{e}}_{i}$ are independent of position, the definition in Equation 72 leads to each basis vector $\boldsymbol{q}_{i}$ expanded in components with respect to the standard Cartesian basis

76 $$\begin{aligned} \boldsymbol{q}_{i} =& \sum _{j} \frac{\partial x^{j}}{\partial q^{i}} \hat{\boldsymbol{e}}_{j}. \end{aligned}$$

In general, the components of the Jacobian are functions of position: $\boldsymbol{q}_{i} = \boldsymbol{q}_{i} ( q^{j} )$.

Moreover, for a given basis $\boldsymbol{q}_{i}$ there exists a unique dual basis $\boldsymbol{q}^{i}$ defined as those linear functionals that satisfy

77 $$\begin{aligned} \boldsymbol{q}^{i} \cdot \boldsymbol{q}_{j} \equiv \delta ^{i}{}_{j}. \end{aligned}$$

(see, *e.g.*, Kobayashi and Nomizu, 1963, pp. 4 – 6 for details). Since the coordinate transformation functions Equation 74 are assumed smooth, that is the Jacobian is locally invertible, the inverse coordinate transformation functions exist. Hence, similar to Equation 76, the dual basis $\boldsymbol{q}^{i}$ may be written in components with respect to the standard Cartesian basis

78 $$\begin{aligned} \boldsymbol{q}^{i} =& \sum _{j} \frac{\partial q^{i}}{\partial x^{j}} \hat{\boldsymbol{e}}_{j}. \end{aligned}$$

In general, the components of the inverse Jacobian are functions of position, $\boldsymbol{q}^{i} = \boldsymbol{q}^{i} ( q^{j} )$.

#### B.1.2 The Metric and the Inner-Product

The standard inner-product between two Cartesian vectors $\boldsymbol{u}$ and $\boldsymbol{u}'$ is a binary operation defined by $\boldsymbol{u} \cdot \boldsymbol{u}' = \sum_{i} u^{i} u^{\prime \,i}$, where $u^{i}$ and $u^{\prime \,i}$ are the components with respect to the standard Cartesian basis. Moreover, the inner-product operation is symmetric [$\boldsymbol{u} \cdot \boldsymbol{u}' = \boldsymbol{u}' \cdot \boldsymbol{u}$], non-degenerate [$\boldsymbol{u} \cdot \boldsymbol{u}' = \boldsymbol{u} \cdot \boldsymbol{u}''$] if and only if $\boldsymbol{u}' = \boldsymbol{u}''$, and positive definite [$\boldsymbol{u} \cdot \boldsymbol{u} \ge 0$] where the equality holds if and only if $\boldsymbol{u} = 0$,

More generally, the inner-product operation between two vectors with components given with respect to a general (curvilinear) basis is defined by a bilinear form $\mathbf{g}$ known as the metric (see, *e.g.*, Kobayashi and Nomizu, 1963, p. 24), $\mathbf{g} ( \boldsymbol{u}, \boldsymbol{u}' ) \equiv \boldsymbol{u} \cdot \boldsymbol{u}'$. The metric $\mathbf{g}$ is symmetric [$\mathbf{g} ( \boldsymbol{u}, \boldsymbol{u}' ) = \mathbf{g} ( \boldsymbol{u}', \boldsymbol{u} )$], non-degenerate [$\mathbf{g} ( \boldsymbol{u}, \boldsymbol{u}' ) = \mathbf{g} ( \boldsymbol{u}, \boldsymbol{u}'' )$], if and only if $\boldsymbol{u}' = \boldsymbol{u}''$, and positive definite [$\mathbf{g} ( \boldsymbol{u}, \boldsymbol{u} ) \ge 0$] where the equality holds if and only if $\boldsymbol{u} = 0$.

Any vector $\boldsymbol{u}$ may be represented as a linear combination of smooth components with respect to the basis $\boldsymbol{q}_{i}$:

79 $$\begin{aligned} \boldsymbol{u} =& \sum_{i} u^{i} \boldsymbol{q}_{i}. \end{aligned}$$

Hence, the inner-product between two vectors with components given with respect to the basis $\boldsymbol{q}_{i}$ is

80 $$\begin{aligned} \boldsymbol{u} \cdot \boldsymbol{u}' =& \mathbf{g} \bigl( \boldsymbol{u}, \boldsymbol{u}' \bigr) = \sum _{i,j} u^{i} u^{\prime \,j} \mathbf{g} ( \boldsymbol{q}_{i}, \boldsymbol{q}_{j} ) = \sum_{i,j} u ^{i} u^{\prime \,j} g_{ij}. \end{aligned}$$

The scalar values $g_{ij} = \mathbf{g} ( \boldsymbol{q}_{i} \boldsymbol{q}_{j} )$, are simply the inner-products between basis vectors $\boldsymbol{q}_{i}$. The $g_{ij}$ may be calculated using Equation 76,

81 $$\begin{aligned} g_{ij} =& \boldsymbol{q}_{i} \cdot \boldsymbol{q}_{j} = \sum_{k,l} \frac{ \partial x^{k}}{\partial q^{i}} \frac{\partial x^{l}}{\partial q ^{j}} ( \hat{\boldsymbol{e}}_{k} \cdot \hat{\boldsymbol{e}}_{l} ) = \sum _{k,l} \frac{\partial x^{k}}{\partial q^{i}} \frac{ \partial x^{l}}{\partial q^{j}} \delta _{kl} = \sum_{k} \frac{ \partial x^{k}}{\partial q^{i}} \frac{\partial x^{k}}{\partial q ^{j}}. \end{aligned}$$

By similar arguments, we may construct (inverse) scalars $g^{ij} = \mathbf{g} ( \boldsymbol{q}^{i}, \boldsymbol{q}^{j} )$ using Equation 78,

82 $$\begin{aligned} g^{ij} =& \boldsymbol{q}^{i} \cdot \boldsymbol{q}^{j} = \sum_{k,l} \frac{ \partial q^{i}}{\partial x^{k}} \frac{\partial q^{j}}{\partial x ^{l}} ( \hat{\boldsymbol{e}}_{k} \cdot \hat{\boldsymbol{e}}_{l} \ ) = \sum _{k,l} \frac{\partial q^{i}}{\partial x^{k}} \frac{ \partial q^{j}}{\partial x^{l}} \delta _{kl} = \sum_{k} \frac{ \partial q^{i}}{\partial x^{k}} \frac{\partial q^{j}}{\partial x ^{k}}. \end{aligned}$$

In general, the scalars $g_{ij} = g_{ij} ( q^{k} )$ and $g^{ij} = g^{ij} ( q^{k} )$, are functions of position. Furthermore, Equations 81 and 82 satisfy

83 $$\begin{aligned} \sum_{k} g_{ik} g^{kj} = \sum_{k} g^{ik} g_{kj} =& \delta ^{i}{}_{j}. \end{aligned}$$

The scalars $g_{ij}$ and $g^{ij}$ may be represented as $3 \times 3$ invertible matrices,

84 $$\begin{aligned} \mathbf{g} =& \sum_{i,j} g_{ij} \boldsymbol{q}^{i} \otimes \boldsymbol{q}^{j}, \quad \mathrm{and} \quad \mathbf{g}^{-1} = \sum _{i,j} g^{ij} \boldsymbol{q}_{i} \otimes \boldsymbol{q}_{j}. \end{aligned}$$

We remark that it is conventional to define the $g_{ij}$ to be the components of the metric $\mathbf{g}$ and the $g^{ij}$ the components of the inverse metric $\mathbf{g}^{-1}$.

By the non-degeneracy assumption, Equations 76, 78, 81, and 82, the metric and inverse metric provide a natural isomorphism between the $\boldsymbol{q}_{i}$ and $\boldsymbol{q}^{i}$. By Equations 82 and 76

85 $$\begin{aligned} \sum_{j} g^{ij} \boldsymbol{q}_{j} =& \sum_{j} \biggl( \sum_{k} \frac{\partial q^{i}}{\partial x^{k}} \frac{\partial q^{j}}{ \partial x^{k}} \biggr) \biggl( \sum_{l} \frac{\partial x^{l}}{ \partial q^{j}} \hat{\boldsymbol{e}}_{l} \biggr) \\ =& \sum_{j,k,l} \frac{\partial q^{i}}{\partial x^{k}} \frac{ \partial q^{j}}{\partial x^{k}} \frac{\partial x^{l}}{\partial q ^{j}} \hat{\boldsymbol{e}}_{l} \\ =& \sum_{k,l} \frac{\partial q^{i}}{\partial x^{k}} \delta _{k} {}^{l} \hat{\boldsymbol{e}}_{l} \\ =& \sum_{k} \frac{\partial q^{i}}{\partial x^{k}} \hat{ \boldsymbol{e}}_{k} \\ =& \boldsymbol{q}^{i}, \end{aligned}$$

where in the last step we used the definition in Equation 78. A similar calculation with Equations 81, 78, and 76 yields

86 $$\begin{aligned} \sum_{j} g_{ij} \boldsymbol{q}^{j} = \boldsymbol{q}_{i}. \end{aligned}$$

#### B.1.3 Curvilinear Coordinates and Orthonormal Bases

On the other hand, one may always construct an orthonormal basis $\hat{\boldsymbol{q}}_{i}$, respectively dual basis $\hat{\boldsymbol{q}} ^{i}$, from linear combinations of the curvilinear coordinate basis $\boldsymbol{q}_{i}$ and dual $\boldsymbol{q}^{i}$ basis,

87 $$ \hat{\boldsymbol{q}}_{i} \equiv \sum _{j} M_{i}{}^{j} \boldsymbol{q} _{j} \quad \mathrm{and} \quad \hat{\boldsymbol{q}}^{i} \equiv \sum_{j} N^{i}{}_{j} \boldsymbol{q} ^{j}. $$

The components of the transformation matrices are smooth functions of the coordinates $M_{i}{}^{j} = M_{i}{}^{j} ( q^{k} )$, respectively $N^{i}{}_{j} ( q^{k} )$. In particular, the matrix components $M_{i}{}^{j}$ and $N^{i}{}_{j}$ are determined by orthonormality conditions,

88 $$\begin{aligned} \begin{aligned} \hat{\boldsymbol{q}}_{i} \cdot \hat{ \boldsymbol{q}}_{j} &\equiv \delta _{ij} = \sum _{k,l} M_{i}{}^{k} M_{j}{}^{l} g_{kl}, \\ \hat{\boldsymbol{q}}^{i} \cdot \hat{\boldsymbol{q}}^{j} &\equiv \delta ^{ij} = \sum_{k,l} N^{i}{}_{k} N^{j}{}_{l} g^{kl}. \end{aligned} \end{aligned}$$

Here the $g_{kl}$ and $g^{kl}$ are given by Equations 81 and 82. Furthermore, orthogonality between the orthonormal basis and dual $\hat{\boldsymbol{q}}_{i} \cdot \hat{\boldsymbol{q}}^{j} = \delta _{i}{} ^{j}$ implies that the transformation matrices are related by $\mathbf{N} = \mathbf{M}^{-1}$.

We remark that by Equations 85 and 86, the coordinate basis $\boldsymbol{q}_{i}$ and dual basis $\boldsymbol{q} ^{i}$ are strictly proportional when these basis sets are *mutually orthogonal*; that is when the metric is diagonal: $\boldsymbol{q}^{i} = g^{ii} \boldsymbol{q}_{i}$ and $\boldsymbol{q}_{i} = g_{ii} \boldsymbol{q}^{i}$. In particular, for *every orthonormal basis set* the basis and dual basis vectors are identified, $\hat{\boldsymbol{q}}^{i} = \hat{\boldsymbol{q}}_{i}$; specifically, the Cartesian basis $\hat{\boldsymbol{e}}^{i} = \hat{\boldsymbol{e}}_{i}$.

#### B.1.4 The Covariant Differential Operator $\nabla $

The covariant differential $\nabla $ is a differential operator that satisfies the standard axioms (see, *e.g.*, Kobayashi and Nomizu, 1963, pp. 123 – 124). In curvilinear coordinates, $\nabla $ is represented with respect to the dual basis $\boldsymbol{q}^{i}$ via its action on smooth functions of position $q^{i}$.

Let $f : \mathbb{R}^{3} \to \mathbb{R}$ be a differentiable scalar function, then the covariant differential of $f$ is

89 $$\begin{aligned} \nabla f =& \sum_{j} ( \nabla _{j} f ) \boldsymbol{q}^{j} = \sum _{j} \frac{\partial f}{\partial q^{j}} \boldsymbol{q}^{j} \end{aligned}$$

(see, *e.g.*, Kobayashi and Nomizu, 1963, p. 143); in Cartesian coordinates the basis and dual are identified, $\hat{\boldsymbol{e}}^{i} = \hat{\boldsymbol{e}}_{i}$, and Equation 89 reduces to the standard gradient. Furthermore, the standard differential is given by

90 $$\begin{aligned} {\mathrm{d}} f =& {\mathrm{d}} \boldsymbol{s} \cdot \nabla f = \sum _{i,j} {\mathrm{d}} q^{i} \,\frac{\partial f}{\partial q^{j}} \bigl( \boldsymbol{q}_{i} \cdot \boldsymbol{q}^{j} \bigr) = \sum_{i} {\mathrm{d}} q^{i}\, \frac{\partial f}{\partial q^{i}}, \end{aligned}$$

where ${\mathrm{d}} \boldsymbol{s} = \sum_{i} {\mathrm{d}} q^{i} \boldsymbol{q}_{i}$, and by Equation 77, $\boldsymbol{q} ^{i} \cdot \boldsymbol{q}_{j} \equiv \delta ^{i}{}_{j}$.

Since the local bases $\boldsymbol{q}_{i}$ are themselves smooth functions of position $q^{i}$, the action of the operator $\nabla $ on the basis vectors is a linear operation,

91 $$\begin{aligned} \nabla \boldsymbol{q}_{i} =& \sum _{j} ( \nabla _{j} \boldsymbol{q}_{i} ) \boldsymbol{q}^{j} = \sum_{j,k} \Gamma ^{k}{}_{ji} \boldsymbol{q}_{k} \otimes \boldsymbol{q}^{j}. \end{aligned}$$

where the linear connection coefficients are functions of position: $\Gamma ^{k}{}_{ji} = \Gamma ^{k}{}_{ji} ( q^{l} )$ (see, *e.g.*, Kobayashi and Nomizu, 1963, p. 143).

A vector field $\boldsymbol{B}$ is a linear combination of smooth component functions $B^{i} = B^{i} ( q^{j} )$ with respect to the basis $\boldsymbol{q}_{i}$:

92 $$\begin{aligned} \boldsymbol{B} =& \sum_{i} B^{i} \boldsymbol{q}_{i}. \end{aligned}$$

The covariant differential $\nabla \boldsymbol{B}$ of the vector field $\boldsymbol{B}$ is then

93 $$\begin{aligned} \nabla \boldsymbol{B} =& \sum_{j} \nabla _{j} \biggl( \sum_{i} B ^{i} \boldsymbol{q}_{i} \biggr) \boldsymbol{q}^{j} \\ =& \sum_{i,j} \bigl( \bigl( \nabla _{j} B^{i} \bigr) \boldsymbol{q}_{i} + B^{i} ( \nabla _{j} \boldsymbol{q}_{i} \ ) \bigr) \boldsymbol{q}^{j} \\ =& \sum_{i,j} \biggl( \frac{\partial B^{i}}{\partial q^{j}} \boldsymbol{q}_{i} + B^{i} \biggl( \sum _{k} \Gamma ^{k}{}_{ji} \boldsymbol{q}_{k} \biggr) \biggr) \boldsymbol{q}^{j} \\ =& \sum_{i,j} \frac{\partial B^{i}}{\partial q^{j}} \boldsymbol{q} _{i} \otimes \boldsymbol{q}^{j} + \sum _{i,j,k} \Gamma ^{i}{}_{jk} B ^{k} \boldsymbol{q}_{i} \otimes \boldsymbol{q}^{j} \\ =& \sum_{i,j} \biggl( \frac{\partial B^{i}}{\partial q^{j}} + \sum _{k} \Gamma ^{i}{}_{jk} B^{k} \biggr) \boldsymbol{q}_{i} \otimes \boldsymbol{q}^{j}, \end{aligned}$$

where we used Equations 89 and 91 in the second step, and we relabeled dummy summation indices in the third step. Hence, the $i$, $j$th component of the matrix representation of $\nabla \boldsymbol{B} ( \boldsymbol{r} )$ with respect to the local basis $\boldsymbol{q}_{i} \otimes \boldsymbol{q}^{i}$ is Equation 21:

$$\begin{aligned} ( \nabla \boldsymbol{B} ){}^{i}{}_{j} =& \frac{\partial B^{i}}{\partial q^{j}} + \sum_{k} \Gamma ^{i}{}_{jk} B^{k} \end{aligned}$$

(see, *e.g.*, Kobayashi and Nomizu, 1963, p. 144).

The matrix representation of the covariant differential with respect to general curvilinear bases explicitly includes terms that describe both the rate of change of the vector components, as well as the manner in which the bases vectors change from point-to-point in the system.

The determination of the linear connection coefficients $\Gamma $ depends on the nature of the basis vectors. In the case of a curvilinear coordinate basis, the linear connection coefficients $\Gamma ^{i}{} _{jk}$ may be written explicitly in terms of coordinate derivatives of the metric

94 $$ \Gamma ^{i}{}_{jk} = \frac{1}{2} \sum_{m} g^{im} \biggl( \frac{ \partial g_{mj}}{\partial q^{k}} + \frac{\partial g_{mk}}{\partial q ^{j}} - \frac{\partial g_{jk}}{\partial q^{m}} \biggr) $$

(see, *e.g.*, Kobayashi and Nomizu, 1963, p. 160).

In the case of an orthonormal basis, the linear connection coefficients $\Gamma ^{i}{}_{jk}$ are given by

95 $$\begin{aligned} \Gamma ^{i}{}_{jk} =& \frac{1}{2} \sum_{m} \delta ^{im} ( c _{kmj} + c_{jmk} - c_{mjk} ), \end{aligned}$$

(see, *e.g.*, Kobayashi and Nomizu, 1963, p. 78). The $c_{ijk}$ in Equation 94 are called *structure constants*, and they are determined by the smooth component functions and derivatives of the transformation matrices in Equation 87; explicitly,

96 $$\begin{aligned} c_{ij}{}^{k} =& \sum _{m,n} \biggl( M_{i}{}^{m} \frac{\partial M _{j}{}^{n}}{\partial q^{m}} \bigl( M^{-1} \bigr)_{n}{}^{k} - M_{j}{}^{n} \frac{ \partial M_{i}{}^{m}}{\partial q^{n}} \bigl( M^{-1} \bigr)_{m}{}^{k} \biggr). \end{aligned}$$

Since, in general, the components and their derivatives of the transformation matrices are functions of position, so also are the structure constants functions of position: $c_{ij}{}^{k} = c_{ij} {}^{k} ( q^{l} )$.

Finally, in the case of the *Cartesian basis*, the linear connection coefficients are all trivial, $\Gamma = 0$, since both the basis and dual basis are independent of position. Therefore, for vectors with components constructed with respect to a Cartesian basis, the covariant differential reduces to the standard partial derivatives with respect to the Cartesian coordinates (see, *e.g.* Equation 15).

### B.2 Spherical–Polar Coordinate Basis

The position of each point $p \in M$ is written in terms of the spherical–polar curvilinear coordinate functions,

97 $$\begin{aligned} \boldsymbol{r} ( p ) =& \bigl\lbrace r ( p ), \theta ( p ), \phi ( p ) \bigr\rbrace \end{aligned}$$

with $r \in [ 0, \infty )$, $\theta \in [ 0, \pi ]$, and $\phi \in [ 0, 2 \pi )$. The Cartesian spherical–polar coordinate transformation functions are

98 $$\begin{aligned} x =& r \sin \theta \cos \phi , \\ y =& r \sin \theta \sin \phi , \\ z =& r \cos \theta . \end{aligned}$$

The position vector is written,

99 $$\begin{aligned} \boldsymbol{r} =& x \hat{\boldsymbol{e}}_{x} + y \hat{ \boldsymbol{e}} _{y} + z \hat{\boldsymbol{e}}_{z} \\ =& ( r \sin \theta \cos \phi ) \hat{\boldsymbol{e}}_{x} + ( r \sin \theta \sin \phi ) \hat{\boldsymbol{e}}_{y} + ( r \cos \theta ) \hat{\boldsymbol{e}}_{z}. \end{aligned}$$

Hence, using the definition of the coordinate basis vectors of Equation 72, the spherical–polar coordinate basis vectors are written in components with respect to the standard Cartesian basis:

100 $$\begin{aligned} \boldsymbol{e}_{r} =& \sin \theta \cos \phi \hat{ \boldsymbol{e}}_{x} + \sin \theta \sin \phi \hat{ \boldsymbol{e}}_{y} + \cos \theta \hat{\boldsymbol{e}}_{z}, \\ \boldsymbol{e}_{\theta } =& r \cos \theta \cos \phi \hat{ \boldsymbol{e}}_{x} + r \cos \theta \sin \phi \hat{ \boldsymbol{e}}_{y} - r \sin \theta \hat{\boldsymbol{e}}_{z}, \\ \boldsymbol{e}_{\phi } =& -r \sin \theta \sin \phi \hat{ \boldsymbol{e}}_{x} + r \sin \theta \cos \phi \hat{ \boldsymbol{e}}_{y}. \end{aligned}$$

Using Equation 81 and the fact that the Cartesian basis are orthonormal, the metric components are immediately identified:

101 $$\begin{aligned} \boldsymbol{e}_{r} \cdot \boldsymbol{e}_{r} = g_{rr} = 1, \qquad \boldsymbol{e}_{\theta } \cdot \boldsymbol{e}_{\theta } = g_{\theta \theta } = r^{2}, \qquad \boldsymbol{e}_{\phi } \cdot \boldsymbol{e}_{\phi } = g_{\phi \phi } = r ^{2} \sin ^{2} \theta . \end{aligned}$$

All other scalar products are zero. Hence, the spherical–polar coordinate basis vectors $\lbrace \boldsymbol{e}_{r}, \boldsymbol{e} _{\theta }, \boldsymbol{e}_{\phi } \rbrace $ are orthogonal, but not orthonormal. Furthermore, the inverse metric components follow immediately:

102 $$\begin{aligned} g^{rr} = 1, \qquad g^{\theta \theta } = \frac{1}{r^{2}}, \qquad g^{\phi \phi } = \frac{1}{r^{2} \sin ^{2} \theta }. \end{aligned}$$

We construct the dual basis using Equation 86 with the inverse metric components of Equation 102,

103 $$\begin{aligned} \boldsymbol{e}^{r} = \boldsymbol{e}_{r}, \qquad \boldsymbol{e}^{\theta } = \frac{\boldsymbol{e}_{\theta }}{r^{2}}, \qquad \boldsymbol{e}^{\phi } = \frac{\boldsymbol{e}_{\phi }}{r^{2} \sin ^{2} \theta }. \end{aligned}$$

Since the metric and inverse components are not orthonormal, the linear connection coefficients are determined by Equation 94. The only non-trivial metric component derivatives are

104 $$\begin{aligned} \frac{\partial g_{\theta \theta }}{\partial r} = 2 r, \qquad \frac{\partial g_{\phi \phi }}{\partial r} = 2 r \sin ^{2} \theta , \qquad \frac{\partial g_{\phi \phi }}{\partial \theta } = 2 r^{2} \sin \theta \cos \theta . \end{aligned}$$

Hence, the only non-trivial linear connection components are

105 $$\begin{gathered} \Gamma ^{r}{}_{\theta \theta } = -r, \qquad \Gamma ^{r}{}_{\phi \phi } = -r \sin ^{2} \theta , \\ \Gamma ^{\theta }{}_{r \theta } = \Gamma ^{\theta }{}_{\theta r} = \frac{1}{r}, \qquad \Gamma ^{\theta }{}_{\phi \phi } = -\sin \theta \cos \theta , \\ \Gamma ^{\phi }{}_{r \phi } = \Gamma ^{\phi }{}_{\phi r} = \frac{1}{r}, \qquad \Gamma ^{\phi }{}_{\theta \phi } = \Gamma ^{\phi }{}_{\phi \theta } = \cot \theta . \end{gathered}$$

Thus, with respect to the spherical–polar curvilinear basis the vector field is

106 $$\begin{aligned} \boldsymbol{B} = B^{r} ( r, \theta , \phi ) \boldsymbol{e} _{r} + B^{\theta } ( r, \theta , \phi ) \boldsymbol{e}_{ \theta } + B^{\phi } ( r, \theta , \phi ) \boldsymbol{e} _{\phi }, \end{aligned}$$

and the matrix representation of the covariant differential $\nabla \boldsymbol{B}$ (Equation 21) is

107 $$\begin{aligned} ( \nabla \boldsymbol{B} ){}^{i}{}_{j} =& \left ( \textstyle\begin{array} {c@{\quad }c@{\quad }c} \frac{\partial B^{r}}{\partial r} & \frac{\partial B^{r}}{\partial \theta } - r B^{\theta } & \frac{\partial B^{r}}{\partial \phi } - r \sin ^{2} \theta B^{\phi } \\ \frac{\partial B^{\theta }}{\partial r} & \frac{\partial B^{\theta }}{\partial \theta } + \frac{B^{r}}{r} & \frac{\partial B^{\theta }}{\partial \phi } - \sin \theta \cos \theta B^{\phi } \\ \frac{\partial B^{\phi }}{\partial r} + \frac{B^{\phi }}{r} & \frac{\partial B^{\phi }}{\partial \theta } + \cot \theta B^{\phi } & \frac{\partial B^{\phi }}{\partial \phi } + \frac{B^{r}}{r} + \cot \theta B^{\theta } \end{array}\displaystyle \right ). \end{aligned}$$

### B.3 Spherical–Polar Orthonormal Basis

By Equation 87, the spherical–polar coordinate basis Equation 100, and the dual basis Equation 103, may be orthonormalized using the transformation matrices $\mathbf{M}$ and $\mathbf{N}$:

108 $$ \mathbf{M} = \left ( \textstyle\begin{array} {c@{\quad}c@{\quad }c} 1 & 0 & 0 \\ 0 & \frac{1}{r} & 0 \\ 0 & 0 & \frac{1}{r \sin \theta } \end{array}\displaystyle \right ), \qquad \mathbf{N} = \mathbf{M}^{-1} = \left ( \textstyle\begin{array} {c@{\quad }c@{\quad}c} 1 & 0 & 0 \\ 0 & r & 0 \\ 0 & 0 & r \sin \theta \end{array}\displaystyle \right ). $$

The spherical–polar orthonromal basis, respectively dual basis, are defined by linear combinations of the spherical–polar coordinate basis $\lbrace \boldsymbol{e}_{r}, \boldsymbol{e}_{\theta }, \boldsymbol{e}_{ \phi } \rbrace $ Equation 100,

109 $$\begin{aligned} \hat{\boldsymbol{e}}_{r} = \boldsymbol{e}_{r}, \qquad \hat{\boldsymbol{e}}_{\theta } = \frac{1}{r} \boldsymbol{e}_{\theta }, \qquad \hat{\boldsymbol{e}}_{\phi } = \frac{1}{r \sin \theta } \boldsymbol{e} _{\phi }, \end{aligned}$$

and the dual basis $\lbrace \boldsymbol{e}^{r}, \boldsymbol{e}^{\theta }, \boldsymbol{e}^{\phi } \rbrace $ Equation 103,

110 $$\begin{aligned} \hat{\boldsymbol{e}}^{r} = \boldsymbol{e}^{r}, \qquad \hat{\boldsymbol{e}}^{\theta } = r \boldsymbol{e}^{\theta }, \qquad \hat{\boldsymbol{e}}^{\phi } = r \sin \theta \boldsymbol{e}^{\phi }. \end{aligned}$$

The transformation matrices, Equation 108, were constructed specifically so that with respect to the basis Equation 109 and dual basis Equation 110, the metric and inverse components are, respectively, $g_{ij} = \delta _{ij}$ and $g^{ij} = \delta ^{ij}$. Hence, the linear connection coefficients are determined by Equation 95. In this case, the non-trivial structure constants determined by Equation 96 are

111 $$\begin{aligned} c_{r \theta \theta } = - c_{\theta r \theta } = c_{r \phi \phi } = - c _{\phi r \phi } = -\frac{1}{r}, \qquad c_{\theta \phi \phi } = -c_{\phi \theta \phi } = - \frac{\cot \theta }{r}, \end{aligned}$$

and the non-trivial linear connection components are

112 $$\begin{aligned} \begin{aligned} \Gamma ^{\theta }{}_{r \theta } &= \Gamma ^{\phi }{}_{r \phi } = - \Gamma ^{r}{}_{\theta \theta } = -\Gamma ^{r}{}_{\phi \phi } = \frac{1}{r}, \\ \Gamma ^{\phi }{}_{\theta \phi } &= -\Gamma ^{\theta }{}_{\phi \phi } = \frac{\cot \theta }{r}. \end{aligned} \end{aligned}$$

Thus, with respect to the spherical–polar orthonormal basis the vector field is

113 $$\begin{aligned} \boldsymbol{B} =& B^{r} ( r, \theta , \phi ) \hat{ \boldsymbol{e}}_{r} + B^{\theta } ( r, \theta , \phi ) \hat{ \boldsymbol{e}}_{\theta } + B^{\phi } ( r, \theta , \phi ) \hat{ \boldsymbol{e}}_{\phi } \end{aligned}$$

and the matrix representation of the covariant differential $\nabla \boldsymbol{B}$ (Equation 21) is

114 $$\begin{aligned} ( \nabla \boldsymbol{B} ){}^{i}{}_{j} =& \left ( \textstyle\begin{array} {c@{\quad }c@{\quad }c} \frac{\partial B^{r}}{\partial r} & \frac{1}{r} \frac{\partial B^{r}}{\partial \theta } - \frac{B^{ \theta }}{r} & \frac{1}{r \sin \theta } \frac{\partial B^{r}}{\partial \phi } - \frac{B ^{\phi }}{r} \\ \frac{\partial B^{\theta }}{\partial r} & \frac{1}{r} \frac{\partial B^{\theta }}{\partial \theta } + \frac{B ^{r}}{r} & \frac{1}{r \sin \theta } \frac{\partial B^{\theta }}{\partial \phi } - \frac{\cot \theta B^{\phi }}{r} \\ \frac{\partial B^{\phi }}{\partial r} & \frac{1}{r} \frac{\partial B^{\phi }}{\partial \theta } & \frac{1}{r \sin \theta } \frac{\partial B^{\phi }}{ \partial \phi } + \frac{B^{r}}{r} + \frac{\cot \theta B^{\theta }}{r} \end{array}\displaystyle \right ). \end{aligned}$$

## Appendix C: On the Determinant of the Matrix Representation of a Smooth, Non-Singular, Linear Map $\mathbf{F}$

In this appendix, we prove Equations 30 and 33 involving the determinant of a square matrix. In particular, given a linear map $\mathbf{F}$, in Appendix C.1 we show that the determinant of the matrix representation of $\mathbf{F}$ with respect to an arbitrary basis is given by the ratio of (signed) differential volume elements in bases related by the action of the transformation $\mathbf{F}$. In Appendix C.2, assuming well-posed ordinary differential equations describing the evolution of the matrix representation of a $C^{n}$ ($n \ge 1$) linear map $\mathbf{F}$, we calculate the rate of change of the determinant with respect to the evolution parameter. Furthermore, this treatment is independent of the basis used to construct the matrix representation of $\mathbf{F}$.

Since we are dealing with the $3 \times 3$ matrix representation of a linear map $\mathbf{F}$, throughout this appendix we make use of the permutation group $S_{3}$ applied to the components of the matrix. The permutation group $S_{3}$ contains $3! = 6$ elements, $S_{3} = \lbrace \sigma _{1}, \dots , \sigma _{6} \rbrace $; written in a tabular format,^7^ we show the action of each element $\sigma _{i} \in S_{3}$ as a column vector

115 $$ \textstyle\begin{array} { c@{\quad } c@{\quad } c@{\quad } c@{ \quad } c@{\quad } c@{\quad } c@{\quad } c@{\quad } c@{\quad } c@{\quad } c@{\quad } c@{\quad } c } S_{3} & | & \sigma _{1} & | & \sigma _{2} & | & \sigma _{3} & | & \sigma _{4} & | & \sigma _{5} & | & \sigma _{6} \\ \hline \sigma _{i} ( 1 ) & | & 1 \to 1 & | & 1 \to 2 & | & 1 \to 1 & | & 1 \to 3 & | & 1 \to 2 & | & 1 \to 3 \\ \sigma _{i} ( 2 ) & | & 2 \to 2 & | & 2 \to 1 & | & 2 \to 3 & | & 2 \to 2 & | & 2 \to 3 & | & 2 \to 1 \\ \sigma _{i} ( 3 ) & | & 3 \to 3 & | & 3 \to 3 & | & 3 \to 2 & | & 3 \to 1 & | & 3 \to 1 & | & 3 \to 2 \end{array} $$

Each $\sigma _{i} \in S_{3}$ may be considered as a function that transposes the labels $( 1, 2, 3 )$. Consider, for example, $\sigma _{2}$ denoted by the second column: $\sigma _{2} ( 1 ) = 2$, $\sigma _{2} ( 2 ) = 1$, and $\sigma _{2} ( 3 ) = 3$. That is, the permutation $\sigma _{2}$ exchanges indices 1 and 2.

The determinant of any $3 \times 3$ matrix may be written in terms of the $S_{3}$ permutation group,^8^

116 $$\begin{aligned} \operatorname{det} \mathbf{F} =& \sum _{\sigma _{i} \in S_{3}} \operatorname{sign} ( \sigma _{i} ) \prod _{j = 1}^{3} F_{j \sigma _{i} ( j )}, \end{aligned}$$

where $\operatorname{sign} : S_{3} \to \lbrace \pm 1 \rbrace $ is the function $\operatorname{sign} ( \sigma _{i} ) = ( -1 ){}^{n}$ such that $n$ is the number of transpositions between adjacent labels needed to return from $\sigma _{i}$ to $\sigma _{1} = ( 1, 2, 3 )$; for example, $\operatorname{sign} ( \sigma _{2} ) = ( -1 ){}^{1} = -1$ since $\sigma _{2} = ( 2, 1, 3 )$ requires one transposition between labels 2 and 1 to match $\sigma _{1} = ( 1, 2, 3 )$. Explicitly, given the labels defined by Equation 115,

117 $$\begin{aligned} \begin{aligned} {\operatorname{sign}} ( \sigma _{1} ) = \operatorname{sign} ( \sigma _{5} ) = \operatorname{sign} ( \sigma _{6} ) &= +1, \\ \operatorname{sign} ( \sigma _{2} ) = \operatorname{sign} ( \sigma _{3} ) = \operatorname{sign} ( \sigma _{4} ) &= -1. \end{aligned} \end{aligned}$$

We note that Equation 116 is independent of the basis in which the matrix $\mathbf{F}$ is represented. Furthermore, the index position in which the pair $( j, \sigma _{i} ( j ) )$ appears is irrelevant.

### C.1 Calculating the Determinant of a Non-Singular, $3 \times 3$ Matrix $\mathbf{F}$ by the Identity Equation 30 in an Arbitrary Basis

Let $( M, h^{i} )$, with $M \subseteq \mathbb{R}^{3}$, be a coordinate chart with an arbitrary basis $\boldsymbol{h}_{i}$. The coordinate differential basis vectors are ${\mathrm{d}} \boldsymbol{h}^{i} = {\mathrm{d}} h^{i} \boldsymbol{h}_{i}$. Written with respect to this basis, the signed differential volume element is

118 $$\begin{aligned} {\mathrm{d}} \Omega ( \boldsymbol{h}_{i} ) =& {\mathrm{d}} \boldsymbol{h}^{3} \cdot \bigl( {\mathrm{d}} \boldsymbol{h}^{1} \times {\mathrm{d}} \boldsymbol{h}^{2} \bigr) \\ =& \bigl( {\mathrm{d}} h^{3} \boldsymbol{h}_{3} \bigr) \cdot \bigl( \bigl( {\mathrm{d}} h^{1}\, \boldsymbol{h}_{1} \bigr) \times \bigl( {\mathrm{d}} h^{2} \,\boldsymbol{h} _{2} \bigr) \bigr) \\ =& \boldsymbol{h}_{3} \cdot ( \boldsymbol{h}_{1} \times \boldsymbol{h}_{2} )\, {\mathrm{d}} h^{1} \, { \mathrm{d}} h^{2}\, {\mathrm{d}} h^{3}. \end{aligned}$$

Suppose the linear map $\mathbf{F}$ may be represented as a non-singular, $3 \times 3$ matrix with respect to this basis $\boldsymbol{h}_{i}$ and dual basis $\boldsymbol{h}{}^{i}$ (see Appendix B.1)

119 $$\begin{aligned} \mathbf{F} = \sum_{i,j} F^{i}{}_{j} \boldsymbol{h}_{i} \otimes \boldsymbol{h}{}^{j}. \end{aligned}$$

Since $\mathbf{F}$ is non-singular, its action is completely described by the transformation of the basis vectors. The propagated coordinate differentials follow immediately:

120 $$\begin{aligned} {\mathrm{d}} \boldsymbol{v}^{i} =& \mathbf{F} \cdot {\mathrm{d}} \boldsymbol{h}^{i} = \sum_{j} F^{j}{}_{i} \,{\mathrm{d}} h^{i}\, \boldsymbol{h}_{j}. \end{aligned}$$

The signed differential volume element with respect to the $\boldsymbol{v}_{i}$ basis is then

121 $$\begin{aligned} {\mathrm{d}} \Omega ( \boldsymbol{v}_{i} ) =& {\mathrm{d}} \boldsymbol{v}^{3} \cdot \bigl( {\mathrm{d}} \boldsymbol{v}^{1} \times {\mathrm{d}} \boldsymbol{v}^{2} \bigr) \\ =& \biggl( \sum_{k} F^{k}{}_{3}\,{\mathrm{d}} h^{3}\, \boldsymbol{h}_{k} \biggr) \cdot \biggl( \biggl( \sum_{i} F^{i}{}_{1} \,{\mathrm{d}} h^{1}\, \boldsymbol{h}_{i} \biggr) \times \biggl( \sum_{j} F^{j}{}_{2} \,{\mathrm{d}} h^{2}\, \boldsymbol{h}_{j} \biggr) \biggr) \\ =& \sum_{i,j,k} F^{i}{}_{1} F^{j}{}_{2} F^{k}{}_{3}\, \boldsymbol{h}_{k} \cdot ( \boldsymbol{h}_{i} \times \boldsymbol{h}_{j} )\, {\mathrm{d}} h^{1}\, { \mathrm{d}} h^{2}\, {\mathrm{d}} h^{3}. \end{aligned}$$

The triple vector product $\boldsymbol{h}_{k} \cdot ( \boldsymbol{h}_{i} \times \boldsymbol{h}_{j} )$ reduces the number of terms in the $i$, $j$, $k$ summation expansion to six; namely, only those involving the permutations of $( i, j, k ) = ( 1, 2, 3 )$ with $i \ne j \ne k$. Hence, we renumerate the reduced $i$, $j$, $k$ summation according to elements of the $S_{3}$ permutation group, Equation 115 (see, *e.g.*, Kobayashi and Nomizu, 1963, p. 283):

122 $$\begin{aligned} {\mathrm{d}} \Omega ( \boldsymbol{v}_{i} ) =& \sum _{\sigma _{i} \in S_{3}} F^{\sigma _{i} ( 1 )}{}_{1} F^{\sigma _{i} ( 2 )}{}_{2} F^{\sigma _{i} ( 3 )} {}_{3}\, \boldsymbol{h}_{\sigma _{i} ( 3 )} \cdot ( \boldsymbol{h}_{\sigma _{i} ( 1 )} \times \boldsymbol{h}_{\sigma _{i} ( 2 )} ) \, {\mathrm{d}} h^{1}\, {\mathrm{d}} h^{2}\, {\mathrm{d}} h^{3} \\ =& \sum_{\sigma _{i} \in S_{3}} F^{\sigma _{i} ( 1 )} {}_{1} F^{\sigma _{i} ( 2 )}{}_{2} F^{\sigma _{i} ( 3 )}{}_{3} \bigl( \operatorname{sign}\, ( \sigma _{i} ) \boldsymbol{h}_{3} \cdot ( \boldsymbol{h} _{1} \times \boldsymbol{h}_{2} ) \bigr) \,{\mathrm{d}} h^{1}\, {\mathrm{d}} h^{2}\, {\mathrm{d}} h^{3} \\ =& \biggl(\, \sum_{\sigma _{i} \in S_{3}} \operatorname{sign} ( \sigma _{i} ) F^{\sigma _{i} ( 1 )}{}_{1} F^{ \sigma _{i} ( 2 )}{}_{2} F^{\sigma _{i} ( 3 )} {}_{3} \biggr) \,{\mathrm{d}} \Omega ( \boldsymbol{h}_{i} ) \\ =& \biggl(\, \sum_{\sigma _{i} \in S_{3}} \operatorname{sign} ( \sigma _{i} ) \prod_{j} F^{\sigma _{i} ( j )}{}_{j} \ \biggr) \, {\mathrm{d}} \Omega ( \boldsymbol{h}_{i} ) \\ =& \operatorname{det} \mathbf{F}\, {\mathrm{d}} \Omega ( \boldsymbol{h}_{i} ), \end{aligned}$$

where we used Equation 118 in the second step.

#### Example

Consider the Cartesian basis $\lbrace \hat{\boldsymbol{e}}_{x}, \hat{\boldsymbol{e}}_{y}, \hat{\boldsymbol{e}} _{z} \rbrace $ with $\hat{\boldsymbol{e}}_{z} \cdot ( \hat{\boldsymbol{e}}_{x} \times \hat{\boldsymbol{e}}_{y} ) = 1$. The differential basis vectors are ${\mathrm{d}} \boldsymbol{x}^{i} = {\mathrm{d}} x^{i} \hat{\boldsymbol{e}}_{i}$. Hence, the initial differential volume element Equation 26 is given by

123 $$\begin{aligned} {\mathrm{d}}\Omega _{0} =& ( \mathrm{d}z\, \hat{ \boldsymbol{e}} _{z} ) \cdot \bigl( ( \mathrm{d}x \,\hat{ \boldsymbol{e}}_{x} ) \times ( \mathrm{d}y \,\hat{\boldsymbol{e}}_{y} ) \bigr) \\ =& \hat{\boldsymbol{e}}_{z} \cdot ( \hat{\boldsymbol{e}}_{x} \times \hat{\boldsymbol{e}}_{y} )\, \mathrm{d}x \mathrm{d}y \, \mathrm{d}z \\ =& \mathrm{d}x\, \mathrm{d}y \,\mathrm{d}z. \end{aligned}$$

Furthermore, the differential volume element Equation 29 propagated along the congruence by the $3 \times 3$ matrix $\mathbf{F}$ is

124 $$\begin{aligned} {\mathrm{d}} \Omega _{\lambda } =& ( \mathbf{F} \cdot { \mathrm{d}} \boldsymbol{z} ) \cdot \bigl( ( \mathbf{F} \cdot {\mathrm{d}} \boldsymbol{x} ) \times ( \mathbf{F} \cdot {\mathrm{d}} \boldsymbol{y} ) \bigr) \\ =& ( \mathrm{d}z \,\mathbf{F} \cdot \hat{\boldsymbol{e}}_{z} ) \cdot \bigl( ( \mathrm{d}x\, \mathbf{F} \cdot \hat{\boldsymbol{e}}_{x} ) \times ( \mathrm{d}y \,\mathbf{F} \cdot \hat{\boldsymbol{e}}_{y} ) \bigr) \\ =& ( \mathbf{F} \cdot \hat{\boldsymbol{e}}_{z} ) \cdot \bigl( ( \mathbf{F} \cdot \hat{\boldsymbol{e}}_{x} ) \times ( \mathbf{F} \cdot \hat{\boldsymbol{e}}_{y} ) \bigr)\, \mathrm{d}x\, \mathrm{d}y\, \mathrm{d}z \\ =& ( \mathbf{F} \cdot \hat{\boldsymbol{e}}_{z} ) \cdot \bigl( ( \mathbf{F} \cdot \hat{\boldsymbol{e}}_{x} ) \times ( \mathbf{F} \cdot \hat{\boldsymbol{e}}_{y} ) \bigr)\, \mathrm{d}\Omega _{0} . \end{aligned}$$

The general $3 \times 3$ matrix components of $\mathbf{F}$ with respect to the (Cartesian) basis $\hat{\boldsymbol{e}}_{i} \otimes \hat{\boldsymbol{e}}^{j}$ are

125 $$ F^{i}{}_{j} = \left ( \textstyle\begin{array} {c@{ \quad }c@{\quad }c} F^{x}{}_{x} &F^{x}{}_{y} & F^{x}{}_{z} \\ F^{y}{}_{x} &F^{y}{}_{y} & F^{y}{}_{z} \\ F^{z}{}_{x} &F^{z}{}_{y} & F^{z}{}_{z} \end{array}\displaystyle \right ). $$

The action of the $3 \times 3$ matrix $\mathbf{F}$ on the basis vectors is

126 $$\begin{aligned} \mathbf{F} \cdot \hat{\boldsymbol{e}}_{i} =& F^{x}{}_{i} \hat{\boldsymbol{e}}_{x} + F^{y}{}_{i} \hat{\boldsymbol{e}}_{y} + F^{z} {}_{i} \hat{\boldsymbol{e}}_{z}. \end{aligned}$$

Then

127 $$\begin{aligned} ( \mathbf{F} \cdot \hat{\boldsymbol{e}}_{x} ) \times ( \mathbf{F} \cdot \hat{\boldsymbol{e}}_{y} ) =& \bigl( F^{y}{}_{x} F^{z}{}_{y} - F^{z}{}_{x} F^{y}{}_{y} \bigr) \hat{\boldsymbol{e}}_{x} \\ +& \bigl( F^{z}{}_{x} F^{x}{}_{y} - F^{x}{}_{x} F^{z}{}_{y} \bigr) \hat{\boldsymbol{e}}_{y} \\ +& \bigl( F^{x}{}_{x} F^{y}{}_{y} - F^{y}{}_{x} F^{x}{}_{y} \bigr) \hat{\boldsymbol{e}}_{z}. \end{aligned}$$

Hence, the triple vector product of the orthonormal Cartesian basis $\boldsymbol{e}_{i}$ under the action of the linear map $\mathbf{F}$ is the determinant of the $3 \times 3$ matrix of $\mathbf{F}$ with the usual meaning,

128 $$\begin{aligned} ( \mathbf{F} \cdot \hat{\boldsymbol{e}}_{z} ) \cdot \bigl( ( \mathbf{F} \cdot \hat{\boldsymbol{e}}_{x} ) \times ( \mathbf{F} \cdot \hat{\boldsymbol{e}}_{y} ) \bigr) =& F^{x}{}_{z} \bigl( F^{y}{}_{x} F^{z}{}_{y} - F ^{z}{}_{x} F^{y}{}_{y} \bigr) \\ +& F^{y}{}_{z} \bigl( F^{z}{}_{x} F^{x}{}_{y} - F^{x}{}_{x} F^{z}{}_{y} \bigr) \\ +& F^{z}{}_{z} \bigl( F^{x}{}_{x} F^{y}{}_{y} - F^{y}{}_{x} F^{x}{}_{y} \bigr) \\ =& \operatorname{det} \mathbf{F}. \end{aligned}$$

Finally, combining Equations 124 and 128, we recover Equation 30.

### C.2 Differentiation of the Determinant of a Smooth, Non-Singular, Linear Map $\mathbf{F} ( \lambda )$

In this appendix we demonstrate Equation 33 for the determinant of a square $3 \times 3$ matrix-valued $C^{n}$ ($n \ge 1$) function of a single real parameter: $\mathbf{F} ( \lambda )$.

Let $\mathbf{F}, \mathbf{M} : \mathbb{R} \to \mathbb{R}^{3 \times 3}$ be smooth, non-singular, linear, $3 \times 3$ matrix-valued functions of $\lambda \in \mathbb{R}$; that is, the components $F_{ij} ( \lambda )$ and $M_{ij} ( \lambda )$ with respect to any basis representation are at least $C^{1}$-functions. Furthermore, assume $\mathbf{F} ( \lambda )$ and $\mathbf{M} ( \lambda )$ satisfy the linear matrix ordinary differential equations for all $\lambda \in \mathbb{R}$; in components with respect to any basis

129 $$\begin{aligned} \frac{\partial F_{ij} ( \lambda )}{\partial \lambda } =& \sum_{k} M_{ik} ( \lambda ) F_{kj} ( \lambda ), \end{aligned}$$

130 $$\begin{aligned} F_{ij} ( 0 ) =& ( F_{0} )_{ij}. \end{aligned}$$

Equations 129 and 130 are a generalization of Equations 16 and 17. In particular, $M_{ij} ( \lambda ) = ( \nabla \boldsymbol{B} \vert _{\boldsymbol{r}} ){}^{i} {}_{j}$ with $\boldsymbol{r} = \boldsymbol{r} ( \lambda , \boldsymbol{r}_{0} )$ and initial condition $( F_{0} )_{ij} = \delta ^{i}{}_{j}$. However, the following results are general, independent of the specifics of the basis representation and physical vector fields.

Writing $\operatorname{det} \mathbf{F}$ with Equation 116, and differentiating with respect to the $\lambda $-parameter,

$$\begin{aligned} \frac{\partial \operatorname{det} \mathbf{F}}{\partial \lambda } =& \frac{ \partial }{\partial \lambda } \biggl( \sum _{\sigma _{i} \in S_{3}} \ \operatorname{sign} ( \sigma _{i} ) \prod_{j} F_{j \sigma _{i} ( j )} \biggr) \\ =& \sum_{\sigma _{i} \in S_{3}} \operatorname{sign} ( \sigma _{i} ) \frac{\partial }{\partial \lambda } ( F_{1 \sigma _{i} ( 1 )} F_{2 \sigma _{i} ( 2 )} F _{3 \sigma _{i} ( 3 )} ) \\ =& \sum_{\sigma _{i} \in S_{3}} \operatorname{sign} ( \sigma _{i} ) \biggl( \frac{\partial F_{1 \sigma _{i} ( 1 )}}{ \partial \lambda } F_{2 \sigma _{i} ( 2 )} F_{3 \sigma _{i} ( 3 )} \biggr) \\ +& \sum_{\sigma _{i} \in S_{3}} \operatorname{sign} ( \sigma _{i} ) \biggl( F_{1 \sigma _{i} ( 1 )} \frac{\partial F_{2 \sigma _{i} ( 2 )}}{\partial \lambda } F_{3 \sigma _{i} ( 3 )} \biggr) \\ +& \sum_{\sigma _{i} \in S_{3}} \operatorname{sign} ( \sigma _{i} ) \biggl( F_{1 \sigma _{i} ( 1 )} F_{2 \sigma _{i} ( 2 )} \frac{\partial F_{3 \sigma _{i} ( 3 )}}{ \partial \lambda } \biggr). \end{aligned}$$

Using Equation 129,

131 $$\begin{aligned} \frac{\partial \operatorname{det} \mathbf{F}}{\partial \lambda } =& \sum_{\sigma _{i} \in S_{3}} \operatorname{sign} ( \sigma _{i} ) \biggl( \sum _{k} M_{1k} F_{k \sigma _{i} ( 1 )} \ \biggr) F_{2 \sigma _{i} ( 2 )} F_{3 \sigma _{i} ( 3 )} \\ +& \sum_{\sigma _{i} \in S_{3}} \operatorname{sign} ( \sigma _{i} ) F_{1 \sigma _{i} ( 1 )} \biggl( \sum_{k} M _{2k} F_{k \sigma _{i} ( 2 )} \biggr) F_{3 \sigma _{i} ( 3 )} \\ +& \sum_{\sigma _{i} \in S_{3}} \operatorname{sign} ( \sigma _{i} ) F_{1 \sigma _{i} ( 1 )} F_{2 \sigma _{i} ( 2 )} \biggl( \sum _{k} M_{3k} F_{k \sigma _{i} ( 3 )} \biggr). \end{aligned}$$

All terms in Equation 131 that are *not* of the form $F_{1 \sigma _{i} ( 1 )} F_{2 \sigma _{i} ( 2 )} F_{3 \sigma _{i} ( 3 )}$ cancel as follows. We may write Equation 131 as

132 $$\begin{aligned} \frac{\partial \operatorname{det} \mathbf{F}}{\partial \lambda } =& \sum_{a, k} C_{ak} M_{ak}, \end{aligned}$$

where the coefficients $C_{ak}$ are defined by

133 $$\begin{aligned} C_{1k} \equiv & \sum_{\sigma _{i} \in S_{3}} \operatorname{sign} ( \sigma _{i} ) F_{k \sigma _{i} ( 1 )} F_{2 \sigma _{i} ( 2 )} F_{3 \sigma _{i} ( 3 )}, \\ C_{2k} \equiv & \sum_{\sigma _{i} \in S_{3}} \operatorname{sign} ( \sigma _{i} ) F_{1 \sigma _{i} ( 1 )} F_{k \sigma _{i} ( 2 )} F_{3 \sigma _{i} ( 3 )}, \\ C_{3k} \equiv & \sum_{\sigma _{i} \in S_{3}} \operatorname{sign} ( \sigma _{i} ) F_{1 \sigma _{i} ( 1 )} F_{2 \sigma _{i} ( 2 )} F_{k \sigma _{i} ( 3 )}. \end{aligned}$$

Each coefficient (Equation 133) is constructed by combinations of the group elements along with their respective sign functions. Then by a judicious regrouping of the summation terms, $C_{ak} = 0$ for $a \ne k$.

For example, consider the coefficient $C_{1k}$ with $k = 2$. Upon expanding the summation, we may regroup the terms by pairing the same value of $\sigma _{i} ( 3 )$ in Equation 115, which defines the ensemble $A_{3} = \lbrace ( \sigma _{1}, \sigma _{2} ), ( \sigma _{6}, \sigma _{3} ), ( \sigma _{5}, \sigma _{4} ) \rbrace $. Then the coefficient $C_{12}$ can be rewritten as

134 $$\begin{aligned} C_{12} \equiv & \sum_{\sigma _{i} \in S_{3}} \operatorname{sign} ( \sigma _{i} ) F_{2 \sigma _{i} ( 1 )} F_{2 \sigma _{i} ( 2 )} F_{3 \sigma _{i} ( 3 )} \\ =& \sum_{ ( \sigma _{i}, \sigma _{j} ) \in A_{3}} ( F_{2 \sigma _{i} ( 1 )} F_{2 \sigma _{i} ( 2 )} - F_{2 \sigma _{j} ( 1 )} F_{2 \sigma _{j} ( 2 )} ) F_{3 \sigma _{i} ( 3 )}, \end{aligned}$$

where the $F_{3 \sigma _{i} ( 3 )}$ term may be factored for each pair, since $\sigma _{i} ( 3 ) = \sigma _{j} ( 3 )$ for all $i, j = 1, 2, 3$. Furthermore, each pair $( \sigma _{i}, \sigma _{j} ) \in A_{3}$ satisfies $\sigma _{i} ( 1 ) = \sigma _{j} ( 2 )$ and $\sigma _{i} ( 2 ) = \sigma _{j} ( 1 )$ (see Table 115). Then the terms in parentheses vanish for each pair $( \sigma _{i}, \sigma _{j} ) \in A_{3}$ and $C_{12} = 0$.

Similarly, for $k = 3$, we have $A_{2} = \lbrace ( \sigma _{1}, \sigma _{4} ), ( \sigma _{6}, \sigma _{2} ), ( \sigma _{5}, \sigma _{3} ) \rbrace $ with $\sigma _{i} ( 2 ) = \sigma _{j} ( 2 )$ for each pair, and

135 $$\begin{aligned} C_{13} =& \sum_{ ( \sigma _{i}, \sigma _{j} ) \in A_{2}} ( F_{3 \sigma _{i} ( 1 )} F_{3 \sigma _{i} ( 3 )} - F_{3 \sigma _{j} ( 1 )} F_{3 \sigma _{j} ( 3 )} ) F_{2 \sigma _{i} ( 2 )} = 0. \end{aligned}$$

The same arguments extend to all $C_{ak}$ for all $a \ne k$, so that only terms $C_{ak}$ for $a = k$ with $F_{1 \sigma _{i} ( 1 )} F_{2 \sigma _{i} ( 2 )} F_{3 \sigma _{i} ( 3 )}$ give non-vanishing contributions. Hence,

136 $$\begin{aligned} \frac{\partial \operatorname{det} \mathbf{F}}{\partial \lambda } =& \sum_{\sigma _{i} \in S_{3}} \operatorname{sign} ( \sigma _{i} ) ( M_{11} + M_{22} + M_{33} ) F_{1 \sigma _{i} ( 1 )} F_{2 \sigma _{i} ( 2 )} F_{3 \sigma _{i} ( 3 )} \\ =& ( M_{11} + M_{22} + M_{33} ) \biggl( \sum _{\sigma _{i} \in S_{3}} \operatorname{sign} ( \sigma _{i} ) F_{1 \sigma _{i} ( 1 )} F_{2 \sigma _{i} ( 2 )} F_{3 \sigma _{i} ( 3 )} \biggr) \\ =& ( \operatorname{Tr} \mathbf{M} ) \biggl( \sum_{\sigma _{i} \in S_{3}} \operatorname{sign} ( \sigma _{i} ) \prod _{j} F_{j \sigma _{i} ( j )} \biggr) \\ =& \operatorname{Tr} \mathbf{M} \operatorname{det} \mathbf{F}. \end{aligned}$$

## Footnotes

1 We remark, unless specifically noted otherwise, throughout this article we work with respect to the standard Cartesian coordinates $\lbrace x, y, z \rbrace $ and orthonormal Cartesian unit basis vectors $\lbrace \hat{\boldsymbol{e}}_{x}, \hat{\boldsymbol{e}}_{y}, \hat{\boldsymbol{e}}_{z} \rbrace $.

2 The function $f$ must be at least $C^{1}$ differentiable. The positivity condition preserves field-line orientation. Furthermore, a solenoidal vector field $\nabla \cdot \boldsymbol{B} = 0$ with $\boldsymbol{X} = \boldsymbol{B} / f$ leads to a constraint $\nabla \cdot \boldsymbol{X} + \boldsymbol{X} \cdot \nabla f = 0$; then choosing a function $f$ such that the vector field $\boldsymbol{X}$ is everywhere tangent to the level surfaces of $f$ (*i.e.* $\boldsymbol{X} \cdot \nabla f = 0$) preserves the solenoidal property to the vector field $\boldsymbol{X}$. Furthermore, including a temporal dependence on $f$ imposes a constraint equation on the $\boldsymbol{X}$-evolution to preserve consistency with $\boldsymbol{B}$.

3 The value of the constant $b$ represents a simple relabeling of the reference point on the particular field-line solution; *i.e.* $\boldsymbol{r}_{0} \mapsto \boldsymbol{r}_{b}$.

4 We reserve the hat-notation for strictly *orthonormal* basis vectors.

5 Compressible vector fields are outside the scope of this work.

6 In solar- and coronal-physics applications, the system boundary is typically taken to be the photosphere-photosphere, or photosphere-source surface ansatz.
